# Circadian‐Related Serotonin/Melatonin Level Modulates Cisplatin Ototoxicity Susceptibility Depended on NOS3–NO Pathway

**DOI:** 10.1111/jpi.70154

**Published:** 2026-06-09

**Authors:** Siyu Qiu, Shiyi Cai, Rong Xie, Chang Liu, Yingzi He

**Affiliations:** ^1^ Department of Otorhinolaryngology, ENT Institute and NHC Key Laboratory of Hearing Medicine, Eye & ENT Hospital Fudan University Shanghai China; ^2^ Institutes of Brain Science, State Key Laboratory of Medical Neurobiology, and MOE Frontiers Center for Brain Science Fudan University Shanghai China; ^3^ Department of Neurosurgery, National Center for Neurological Disorders, Neurosurgical Institute of Fudan University, Shanghai Clinical Medical Center of Neurosurgery, Shanghai Key Laboratory of Brain Function and Restoration and Neural Regeneration, Huashan Hospital Fudan University Shanghai China; ^4^ Department of Otolaryngology–Head and Neck Surgery The Third Affiliated Hospital of Sun Yat‐Sen University Guangzhou Guangdong China

**Keywords:** circadian rhythm, CREB, melatonin, NO, NOS3, serotonin

## Abstract

Hearing impairment is attributed to factors such as age, genetic predisposition, and environmental influences, among which environmental factors are considered modifiable. Among various environmental factors, the role of poor lifestyle habits is particularly critical, yet the specific mechanisms by which they contribute to hearing damage remain unclear. This study reveals that dysregulated hormone levels due to disrupted light exposure may significantly increase susceptibility to sensorineural hearing loss. In mice, circadian rhythm disruption was found to reduce melatonin and elevate serotonin levels in the inner ear, thereby increasing vulnerability to cisplatin‐induced ototoxicity. In both in vivo and in vitro cisplatin‐treatment models, we showed that combined treatment with melatonin protected hearing, reduced inner ear cell death, and preserved synaptic connections, whereas serotonin co‐administration exacerbated the damage. Using small molecule–protein interaction prediction, we identified NOS3 as a potential target of both melatonin and serotonin, through which they appear to regulate the NO signaling pathway and influence hair cell ferroptosis. Finally, exogenous supplementation of NOS3 in cochlear tissues effectively mitigated cisplatin‐induced hair cell damage, even under conditions of circadian rhythm disruption. These findings indicate that the melatonin/serotonin balance modulates susceptibility to sensorineural hearing loss via the NOS3–NO signaling pathway.

## 1 Introduction

Of the global population, about 430 million people, or over 5%, live with disabling hearing loss (WHO, 2025). This number is estimated to exceed 500 million (one in 10 people worldwide) by 2030. Many causes of hearing loss can be prevented through public health strategies and clinical interventions implemented across the lifespan, including avoiding excessive noise exposure and judicious use of ototoxic medications. However, beyond these established environmental and pharmacological factors, whether long‐term individual lifestyle habits, for example, those related to diet and sleep, are independent risk factors remains unclear.

Multiple studies have demonstrated that susceptibility to sensorineural hearing loss fluctuates with circadian rhythms. Nighttime noise exposure causes more pronounced hearing threshold shifts than morning exposure, following noise‐induced hearing loss. This difference may be linked to circadian variations in glucocorticoid secretion, the expression of clock genes and their targets, and immune system regulation [1, 2, 3, 4]. Circadian rhythm disruption is increasingly implicated in adverse prognostic outcomes for sensorineural hearing loss. This is evidenced by reduced peripheral expression of circadian clock genes in patients with sudden hearing loss, particularly when accompanied by vertigo. Moreover, the degree of recovery shows only a weak association with the restoration of normal gene expression levels following treatment [5, 6]. Furthermore, experimental disruption of circadian rhythms through altered lighting conditions exacerbates noise‐induced hearing loss and elevates oxidative stress in the mouse cochlea [7]. In summary, the available evidence points to the potential of circadian rhythm regulation as a viable target, offering a novel strategy for intervention in sensorineural hearing loss.

Research indicates a bidirectional relationship between sleep and cancer. Sleep disturbances in cancer patients are primarily associated with the activation of systemic inflammatory responses. Tumor tissues spontaneously or reactively release pro‐inflammatory cytokines, which further affect the suprachiasmatic nucleus (SCN) and ventrolateral preoptic area (VLPO) of the hypothalamus, leading to sleep disorders. Additionally, tumor‐related hormonal alterations and metabolic disturbances further activate orexin neurons and brainstem arousal circuits, exacerbating sleep fragmentation and circadian rhythm disruption [8]. This disruption of circadian rhythms not only affects disease progression but also directly interferes with the efficacy of cancer treatments. For example, the toxicity of the widely used chemotherapeutic agent cisplatin is closely linked to circadian biology. Studies have shown that cisplatin‐induced ototoxicity exhibits circadian sensitivity, with administration during the active phase mitigating hearing damage. However, no studies to date have investigated the relationship between circadian rhythm disruption and the prognosis of cisplatin‐induced ototoxicity [9, 10].

Numerous studies have demonstrated that the endogenous circadian clock regulates physiological functions through hormones, while circadian rhythm disruption is strongly associated with dysregulated hormone secretion [1, 11]. For instance, adrenalectomy—which removes circulating glucocorticoids—has been shown to markedly suppress the transcription of clock‐controlled genes involved in inflammatory responses, without affecting cochlear clock rhythms. Furthermore, exogenous administration of dexamethasone provided effective protection against acute noise‐induced injury only when administered during the daytime, when endogenous glucocorticoid levels are low. Together, these findings highlight the critical role of endocrine hormones in governing circadian auditory sensitivity and pave the way for optimizing sensorineural hearing loss treatment through circadian rhythm‐based therapeutic strategies.

Melatonin is an endocrine hormone closely associated with sleep, and its processes of synthesis, storage, and secretion inherently demonstrate a clear circadian regulatory capacity. It is synthesized and stored in the pineal gland and secreted specifically at night, promoting sleep by inhibiting central nervous system excitability. Importantly, the metabolism and release of melatonin and its precursor serotonin are precisely regulated by sympathetic nerve activity, retinal light signal input, and intrinsic negative feedback mechanisms, resulting in typical circadian rhythm variations in their levels and maintaining a dynamic balance [12, 13, 14]. This regulatory system forms the basis for its core physiological functions.

Besides its core role in regulating biological rhythms, melatonin also acts as a highly effective and low‐toxicity antioxidant. In this capacity, it alleviates oxidative stress in inner ear cells, regulates potassium ion channels and currents, suppresses inflammatory responses and various forms of programmed cell death, and mitigates hair cell damage induced by age‐related hearing loss, noise, ototoxic drugs, radiation, and inner ear hemorrhage [15, 16, 17, 18, 19, 20, 21]. Relatedly, its precursor serotonin functions as a neuromodulator, coordinating multisensory signaling networks, including the auditory pathway, and influencing mechanical frequency tuning [22, 23]. In neomycin‐induced ototoxicity models, tryptophan hydroxylase‐mediated serotonin biosynthesis exacerbates apoptosis in cochlear hair cells and spiral ganglion neurons [24]. However, whether circadian rhythms modulate the melatonin/serotonin homeostasis underlying the circadian susceptibility to cisplatin‐induced ototoxicity remains to be elucidated. In sight, pharmacological interventions targeting modifiable risk factors—hormone imbalance—offer a complementary strategy for ototoxicity [25].

Given that the causal relationship between circadian rhythm disruption and cisplatin‐induced ototoxicity—particularly the underlying key biochemical mechanisms—remains unclear, we established a corresponding mouse model for investigation. This model revealed that circadian disruption specifically impairs the biosynthesis of melatonin from serotonin in the inner ear, leading to a hormonal imbalance characterized by a deficiency in protective melatonin and an accumulation of serotonin. This imbalance was closely associated with exacerbated hearing loss, increased hair cell death, and synaptic damage. Both in vivo and in vitro validation experiments demonstrated that exogenous melatonin counteracts cisplatin‐induced ototoxicity, whereas serotonin supplementation aggravates the injury. To elucidate the downstream molecular mechanisms of this hormonal imbalance, we integrated bioinformatics predictions with transcriptomic data and further verified the findings through molecular docking. Ultimately, we identified NOS3 as a central mediator of both the protective effects of melatonin and the toxic effects of serotonin. In the explant model, cisplatin downregulated the expression of Nitric Oxide Synthase 3 (NOS3)/p‐NOS3 and reduced nitric oxide (NO) levels, thereby inducing ferroptosis. Melatonin reversed these alterations, while serotonin exacerbated them. Importantly, in the animal model, exogenous restoration of NOS3 effectively alleviated hair cell death, synaptic loss, oxidative stress, and ferroptosis, thereby preserving auditory function. These findings delineate a pathogenic cascade that originates from circadian rhythm disruption and involves impaired inner ear melatonin synthesis, a consequent dysregulated melatonin/serotonin ratio, inactivation of the NOS3–NO signaling axis, oxidative stress with ferroptosis, and ultimately apoptosis of hair cells and neurons leading to hearing loss. This provides a solid theoretical basis for preventing cisplatin‐induced ototoxicity by correcting the core biochemical conversion defect, restoring hormonal balance, and targeting NOS3 activation.

## 2 Materials and Methods

### 2.1 Animals

Wild‐type C57BL/6 mice used in this study were obtained from Jihui Biotechnology Co. Ltd., and housed in a specific pathogen‐free facility. All animals were maintained under the same dietary conditions with free access to food and water and were allowed to move freely within their cages. The housing environment was maintained at constant temperature and humidity under a 12‐h light/12‐h dark cycle, with lights on from 07:00 (designated as zeitgeber time 0, ZT0) to 19:00 (ZT12). All animal experiments and procedures were performed in accordance with guidelines approved by the Shanghai Medical Laboratory Animal Management Committee and the Animal Ethics Committee of Fudan University.

### 2.2 In Vivo Experiment

To evaluate the effects of circadian intervention during cisplatin chemotherapy, we established a corresponding mouse model based on established protocols [7, 26]. Six‐week‐old mice were acclimated for 2 weeks under a standard 12‐h light/12‐h dark cycle. They were then randomly assigned to three groups for a 1‐week light/dark regime pretreatment: Light–Dark (LD), continuous light (LL), or continuous dark (DD). Following pretreatment, all mice underwent three cycles of cisplatin treatment. Each cycle consisted of intraperitoneal injections at a dose of 3.5 mg/kg/day for four consecutive days, followed by a 10‐day drug‐free recovery period.

To assess the potential protective or aggravating effects of melatonin and serotonin on cisplatin‐induced ototoxicity, mice were acclimatized for 2 weeks under standard conditions and then randomly divided into four groups: (1) Control (saline), (2) Cisplatin alone, (3) Cisplatin + Melatonin, and (4) Cisplatin + Serotonin. All groups except Control received three cycles of cisplatin (3.5 mg/kg/day, i.p.; Sigma‐Aldrich, 15663‐27‐1). Two hours prior to each cisplatin injection, the following pretreatments were administered intraperitoneally: saline to the Control and Cisplatin alone groups, melatonin (20 mg/kg; MedChemExpress, HY‐B0075) to the Cisplatin + Melatonin group, and serotonin (20 mg/kg; MedChemExpress, HY‐B1473A) to the Cisplatin + Serotonin group.

Following the circadian intervention (LD, LL, or DD) as previously described, mice were randomly assigned to three groups: (1) Control, (2) Cisplatin alone, and (3) Cisplatin + NOS3. One day prior to the initiation of cisplatin chemotherapy, mice in the Control and Cisplatin alone groups received an injection of simulated perilymphatic fluid (via the posterior semicircular canal, PSC), whereas mice in the Cisplatin + NOS3 group received 2 µg of recombinant NOS3 protein (CUSABIO, CSB‐EP015946MO) via the same route. All groups except the Control subsequently underwent three cycles of cisplatin administration (3.5 mg/kg/day, i.p.).

### 2.3 Auditory Function Testing

Mice were anesthetized via intraperitoneal injection of a mixture of tiletamine‐zolazepam (50 mg/kg, Zoletil50, Virbac) and dexmedetomidine (50 mg/kg, Orion). Auditory tests were conducted inside a sound‐attenuating chamber using a TDT BioSigRZ system (Tucker–Davis Technologies).

Auditory Brainstem Response (ABR): Tone bursts (4–32 kHz) were delivered via a free‐field speaker. Stimulus intensity decreased from 90 dB SPL in 5‐dB steps, with 400 averages recorded per intensity level. Subcutaneous needle electrodes were placed at the vertex (active), ipsilateral mastoid (reference), and hip (ground). The ABR threshold for each frequency was defined as the lowest intensity that elicited a reproducible Wave II.

Distortion Product Otoacoustic Emission (DPOAE): Two primary tones (f1 and f2, with f2/f1 ratio = 1.2, f2 ranging from 4 to 32 kHz) were presented via an ear‐probe speaker system. Stimulus levels (L1 = L2) decreased from 80 dB SPL in 5‐dB steps. The cubic distortion product at the frequency 2f1–f2 was recorded using a probe microphone. The DPOAE threshold was defined as the lowest stimulus level that produced a response at least 3 dB above the noise floor.

### 2.4 Preparation and Treatment of Cochlear Explants

Cochlear explants were prepared under sterile conditions from postnatal day 2–3 (P2–P3) mice housed under specific pathogen‐free (SPF) conditions. Following decapitation, the skull was bisected sagittally. Temporal bones were isolated, briefly immersed in 75% ethanol for disinfection, and then thoroughly rinsed in ice‐cold phosphate‐buffered saline (PBS).

All subsequent dissections were performed under a stereomicroscope with the tissues immersed in ice‐cold PBS. The cochlea was carefully exposed. The spiral ligament was meticulously removed while avoiding contact with the underlying basilar membrane. For basilar membrane explants, the membrane was gently separated from the modiolus. For spiral ganglion neuron (SGN) explants, the middle turn of the cochlea along with the attached modiolus was excised.

The isolated tissues were transferred onto glass coverslips pre‐coated with Cell‐Tak (Corning, 354240). Explants were oriented with the vestibular membrane facing upward to facilitate flattening and cultured in DMEM/F12 medium (HyClone, SH30023.01) supplemented with 1% N2 supplement (Thermo Fisher Scientific, TM17502048), 2% B27 supplement (Thermo Fisher Scientific, TM17504044), and 2% ampicillin (Sangon Biotech, B540722). Cultures were maintained at 37°C in a humidified atmosphere of 5% CO_2_.

After a 4–6‐h attachment period (designated as time 0 h), the explants were treated for 24 h with culture medium containing cisplatin and/or other experimental agents. Subsequently, the treatment medium was replaced with fresh standard explant medium, and the cultures were maintained until collection at the designated experimental endpoints.

### 2.5 Immunofluorescence Staining

Immunofluorescence was conducted on two types of cochlear specimens: (A) tissue sections or cultured explants, and (B) whole‐mount cochlear preparations.

A. Staining of Tissue Sections and Cochlear Explants
  Decalcified adult mouse basilar membranes or explants cultured for 72–96 h were washed with PBS and fixed in 4% paraformaldehyde for 30 min at room temperature. After permeabilization and blocking in PBS containing 1% Triton X‐100 and 10% fetal bovine serum (1% PBST + 10% FBS) for 1 h, samples were incubated overnight at 4°C with primary antibodies diluted in 1% PBST. The primary antibodies used were: rabbit anti‐Myosin VIIa (1:500, bs‐7761 R‐HRP, Proteus BioSciences), chicken anti‐Neurofilament (1:500, ab254348, Abcam), goat anti‐SOX2 (1:500, AF2018, R&D Systems), mouse anti‐β‐III‐tubulin (Tuj1, IgG2a; 1:500, 801202, BioLegend), rabbit anti‐Homer1 (1:500, ab184955, Abcam), and mouse anti‐CtBP2 (IgG1; 1:500, 612044, BD Biosciences). Following three washes with PBS, samples were incubated for 2 h at room temperature in the dark with appropriate Alexa Fluor 488‐ or Cy3‐conjugated donkey secondary antibodies (Invitrogen) diluted in 1% PBST. Nuclei were counterstained with DAPI (1:1000, 62248, Thermo Fisher Scientific).
B. Staining of Whole‐Mount Cochleae
  For whole‐mount preparations, mice were anesthetized and decapitated, and the cochleae were rapidly dissected and fixed in 4% paraformaldehyde overnight at 4°C. Fixed cochleae were decalcified in 120 mM EDTA (pH 7.4) for 3–4 days at 4°C. After decalcification, the basilar membranes were micro‐dissected. Tissues were then permeabilized and blocked as described in section A (1% PBST + 10% FBS). Primary antibody incubation was performed overnight at 4°C using the following antibodies diluted in 1% PBST: rabbit anti‐Myosin VIIa (1:500, Proteus Biosciences), goat anti‐Prestin (1:500, sc‐22692, Santa Cruz), chicken anti‐Neurofilament (1:500, Abcam), rabbit anti‐Homer1 (homer scaffold protein 1, (1:500, 160 003, Synaptic Systems), and mouse anti‐CtBP2 (C‐terminal‐binding protein 2, (1:500, BD Biosciences). For F‐actin staining, samples were incubated with Alexa Fluor 488‐phalloidin (1:500, A12379, Thermo Fisher Scientific) for 30 min at room temperature in the dark. After washing, appropriate Alexa Fluor 488‐, 568‐, or 647‐conjugated secondary antibodies were applied for 2 h at room temperature. Nuclei were counterstained with DAPI (4′,6‐diamidino‐2‐phenylindole) (1:1000, D9542, Sigma‐Aldrich).
C. Mounting and Imaging

For both protocols, after final washes in PBS, all stained samples were mounted in Fluoromount‐G mounting medium and coverslipped. Images were acquired using a Leica STELLARIS 8 STED laser‐scanning confocal microscope equipped with appropriate laser lines and filter sets.

### 2.6 Image Processing and Quantitative Analysis

Z‐stack images were processed and merged using ImageJ (National Institutes of Health, Bethesda, MD, USA). Quantitative analyses included the following measurements performed on the processed images: the number of morphologically intact Myosin VIIa‐positive hair cells per 100 μm length of the organ of Corti; the number of Homer1/Ctbp2‐double‐positive puncta per inner hair cell; the density of SOX2‐positive supporting cells per 10 000 μm^2^ within the sensory epithelium; the density of Neurofilament‐ or Tuj1‐positive nerve fibers per 100 μm length; and the density of Tuj1‐positive spiral ganglion neuron somata per 10 000 μm^2^ in the modiolar region. Finally, micrographs were cropped to appropriate dimensions using Adobe Photoshop and assembled for presentation using Adobe Illustrator (both Adobe Inc., San Jose, CA, USA).

### 2.7 Reactive Oxygen Species‌ (ROS) and Lipid Peroxidation Detection

At 48 h of culture, basilar membrane explants were incubated with MitoSOX Red (1:2000, M36008, Invitrogen) and BODIPY 493/503 methyl bromide (1:5000, HY‐D1614, MedChemExpress) for 30 min at 37°C in the dark. Following PBS washes and fixation, F‐actin was labeled with Alexa Fluor 647‐phalloidin (1:40) for 30 min, and nuclei were counterstained with DAPI. Samples were mounted and imaged using a 40× oil‐immersion objective on a confocal microscope (1024 × 1024 pixel resolution, 2 µm z‐step size). BODIPY, MitoSOX Red, and phalloidin signals were captured using the 488, 555, and 647 nm channels, respectively. Mean fluorescence intensities for BODIPY (reflecting lipid peroxidation) and MitoSOX Red (indicating ROS levels) were quantified within phalloidin‐demarcated hair cell regions using ImageJ software (National Institutes of Health) and compared to control samples.

### 2.8 Fe^2+^ Detection

Basilar membrane explants were incubated after 48 h of culture with FerrOrange (F374, Dojindo) and Mito FerroGreen (M489, Dojindo) for 30 min at 37°C in the dark. Following washing and fixation, F‐actin and nuclei were labeled with phalloidin and DAPI, respectively. Signals from Mito FerroGreen (excitation 488 nm) and FerrOrange (excitation 555 nm) within the phalloidin‐demarcated hair cell region were quantified using ImageJ. The mean fluorescence intensities, corresponding to mitochondrial Fe^2+^ and total intracellular Fe^2+^ levels, respectively, were normalized and compared with those of the control group.

### 2.9 NOS3 Level Measurement

Basilar membrane explants were fixed and permeabilized after 48 h of culture and then incubated overnight at 4°C with the following primary antibodies: mouse anti‐parvalbumin (1:500, ab277625, Abcam), rabbit anti‐NOS3 (AF0096, Affinity Biosciences), and rabbit anti‐NOS3 (phospho‐Ser1177) (AF3247, Affinity Biosciences). After PBS washes, corresponding species‐specific fluorescent secondary antibodies were applied for 2 h at room temperature. Nuclei were counterstained with DAPI. The parvalbumin signal was used to delineate hair cell regions. Within these regions, the mean fluorescence intensities of NOS3 and phospho‐NOS3 (p‐NOS3) were quantified using ImageJ, normalized as appropriate, and compared with those of the control group.

### 2.10 Ferroptosis Detection

At 48 h, explants were fixed, permeabilized, blocked, and incubated overnight at 4°C with primary antibodies against Parvalbumin (MA5‐47410, Invitrogen) and Transferrin Receptor (TfR, ab84036, Abcam). After washing, fluorescent secondary antibodies were applied for 2 h. Nuclei were stained with DAPI. The number of Parvalbumin‐positive hair cells and Parvalbumin/Transferrin Receptor double‐positive ferroptotic hair cells per 100 µm along the Corti's organ was quantified.

### 2.11 Measurement of Melatonin and Serotonin

Blood was collected by cardiac puncture and centrifuged to separate serum. Cochleae were dissected and homogenized in RIPA lysis buffer (P0013B, Beyotime). Basilar membrane explants were washed, transferred to lysis buffer, and subjected to sonication. All samples were centrifuged, and the supernatants were collected for subsequent analysis.

Melatonin and serotonin levels in serum and cochlear lysates were measured using ELISA kits (melatonin: ml077242; serotonin: ml023035; Enzyme‐linked Biotechnology) according to the manufacturer's instructions, with serial dilutions of standards. After incubation with detection antibody and streptavidin‐HRP, absorbance was measured at 450 nm. Nitric oxide in basilar membrane lysates was quantified with a Griess assay kit (SB‐JK1030, Share‐Bio), and absorbance was read at 540 nm. Analyte concentrations were calculated from their respective standard curves and corrected for dilution factor or tissue weight as appropriate.

### 2.12 Western Blot Analysis

Western blot analysis was performed as follows: at 24 h, protein was extracted from explants by lysis in ice‐cold RIPA buffer, followed by sonication (90 W, 0.2 s on/1.0 s off, 30 s total) and centrifugation (12 000 rpm, 15 min, 4°C). The supernatant was denatured in SDS–PAGE sample buffer (08W00015; MP Biomedicals, Santa Ana, CA, USA), separated on a 12% SDS–PAGE gel (150 V, 45 min), and transferred to a PVDF membrane (IPVH00010; Merck Millipore, Zug, Switzerland) at 400 mA for 60 min in pre‐cooled transfer buffer. After blocking with QuickBlock Blocking Buffer (P0235, Beyotime), the membrane was incubated overnight at 4°C with primary antibodies diluted in QuickBlock Antibody Dilution Solution (P0023A, Beyotime), including rabbit anti‐4‐HNE (1:750, ab46545; Abcam), anti‐LPO (ab166615; Abcam), anti‐GPX4 (1:750, ab125066; Abcam), anti‐SLC7A11 (1:750, 12691; Cell Signaling Technology), anti‐p53 (1:750, 9282; Cell Signaling Technology), anti‐FTH1 (1:750, 4393; Cell Signaling Technology), and mouse anti‐GAPDH (1:5000, 437000; Invitrogen). After washing, the membrane was incubated for 90 min at room temperature with an HRP‐conjugated donkey anti‐rabbit secondary antibody (406401; BioLegend) diluted in TBST. Signals were detected using an enhanced chemiluminescence kit (P0018FM, Beyotime), imaged with a ChemiDoc MP system, and quantified with ImageJ software after normalization to GAPDH.

### 2.13 Quantitative PCR Array

Total RNA was extracted from basilar membrane explants at 24 h using TRIzol reagent (15596018, Invitrogen), followed by chloroform separation and isopropanol precipitation. The RNA pellet was washed with 75% (v/v) ethanol, air‐dried, and dissolved in DEPC‐treated water. Genomic DNA was eliminated, and reverse transcription was performed using the PrimeScript RT Reagent Kit with gDNA Eraser (RR047A, Takara). Quantitative PCR was conducted using a commercial PCR array focused on the nitric oxide signaling pathway (wc‐mRNA0108‐M, WCGENE BIOTECH). The thermal cycling protocol consisted of initial denaturation at 95°C for 3 min, followed by 40 cycles of 95°C for 10 s and 60°C for 30 s, with a subsequent melt curve analysis. Relative gene expression was calculated using the 2^−ΔΔCT^ method, normalized to housekeeping genes, and compared to the control group.

### 2.14 Phosphoprotein Profiling Array

Phosphorylation profiling was performed using a commercial antibody array. Briefly, explants were harvested at 48 h, lysed in RIPA buffer, sonicated on ice, and centrifuged. The supernatant was collected and stored at −80°C until use. A mouse phosphorylation multi‐pathway profiling array kit (AAM‐PPP‐1, RayBiotech) was used according to the manufacturer's instructions. Membranes were blocked and then incubated with samples overnight at 4°C. After washing, membranes were incubated with a detection antibody cocktail overnight, followed by an HRP‐conjugated secondary antibody for 2 h at room temperature. Signals were developed with a chemiluminescent substrate and captured using a chemiluminescence imaging system. Spot intensities were quantified, normalized to internal positive controls, and compared to the control group.

### 2.15 Statistical Analysis

All data were analyzed using GraphPad Prism 8 (GraphPad Software, San Diego, CA, USA) and are expressed as means ± SEM. For most experiments, statistical significance was assessed by one‐way ANOVA followed by Dunnett's post hoc test. Auditory brainstem response (ABR) threshold data, which involved two independent factors (group and frequency), were analyzed by two‐way ANOVA with Tukey's multiple comparisons test. For multiple comparisons following one‐way ANOVA, Bonferroni post hoc correction was applied to control the family‐wise error rate. For two‐way ANOVA (ABR threshold analysis), Šidák or Bonferroni correction was used for multiple comparisons across frequency and group. A *p*‐value < 0.05 was considered statistically significant.

## 3 Results

### 3.1 Circadian Disruption Alters Melatonin/Serotonin Levels in the Cochlea and Serum

To disrupt circadian rhythms, mice were housed under altered lighting conditions. Compared with the standard light–dark cycle group, both constant light and constant dark conditions abolished the rhythmic expression of core circadian clock genes, which is consistent with previous reports (Supplementary Figure 1) [1, 7, 11].

**FIGURE 1:**
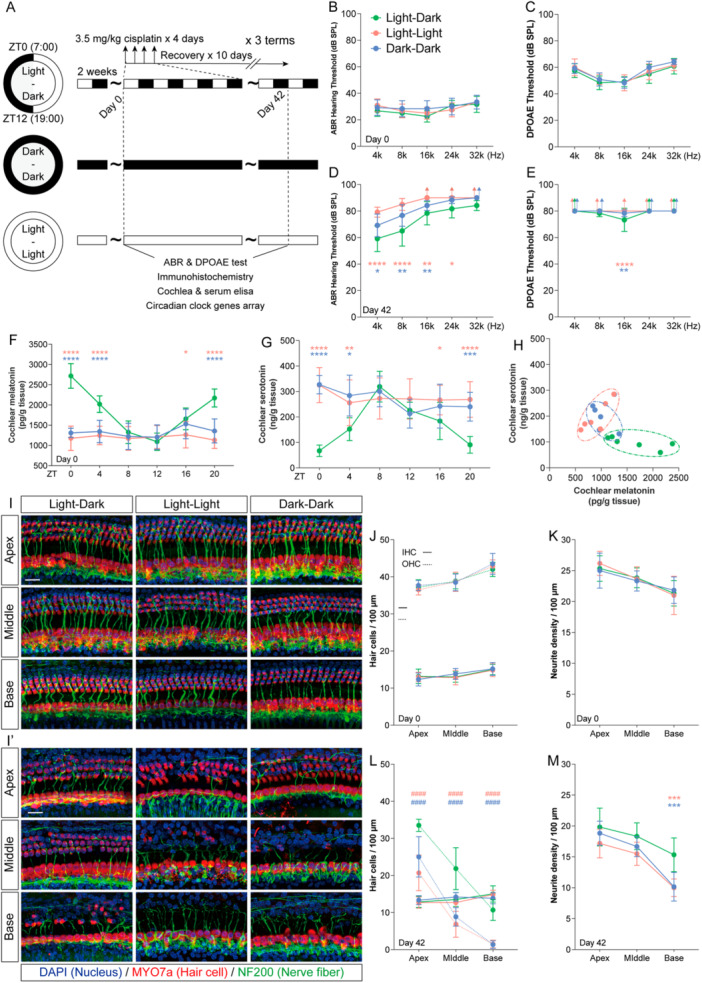
Circadian disruption exacerbates cisplatin‐induced ototoxicity. (A) Schematic of the experimental design combining circadian disruption and cisplatin chemotherapy in mice. (B–E) ABR and DPOAE thresholds before chemotherapy (Day 0) and after chemotherapy (Day 42). (F and G) Circadian profiles of cochlear melatonin (F) and serotonin (G) concentrations at 6 Zeitgeber times (ZTs) on Day 0. (H) Cochlear melatonin and serotonin concentrations at ZT0 on Day 42 after chemotherapy. (I and I′) Representative immunofluorescence images of apical, middle, and basal turns of the cochlear basilar membrane on Day 0 (I) and Day 42 (I′). Myosin 7a (red) labels hair cells, Neurofilament (green) labels auditory nerve fibers, and DAPI (blue) stains nuclei. Scale bars = 20 μm (J and K) Quantitative analysis of inner hair cell (solid lines) and outer hair cell (dashed lines) densities in the apical, middle, and basal turns on Day 0 (J) and Day 42 (K). No significant difference was observed in inner hair cell density among groups. ^####^ *p* < 0.0001 indicates the comparison of outer hair cell density between each group and the Light–Dark (LD) group (*n* = 6). (L and M) Quantitative analysis of auditory nerve fiber density and length in the apical, middle, and basal turns on Day 0 (L) and Day 42 (M). Data are presented as mean ± SEM. ****p < 0.0001, ***p < 0.001, **p < 0.01, *p < 0.05 vs. the LD group; one‐way ANOVA with Bonferroni post hoc test, *n* = 6.

Previous studies indicate that individuals with sleep disorders exhibit distinct metabolic profiles and genetic polymorphisms. Notably, many of the differential metabolites and susceptibility genes identified are associated with the metabolism and signaling pathways of melatonin and serotonin [27, 28]. Therefore, we measured melatonin and serotonin levels in the cochlea and serum. Under normal light–dark (Light–Dark) conditions, both serum and inner ear tissues exhibited circadian variations in melatonin and serotonin levels: during the light phase, melatonin levels decreased while serotonin levels increased; during the dark phase, melatonin levels increased while serotonin levels decreased. In contrast, mice exposed to constant light (Light–Light), or constant dark (Dark–Dark) conditions showed no circadian rhythm in melatonin or serotonin levels in either serum or inner ear tissues. Circadian disruption‐maintained melatonin at a persistently low level, near its physiological trough, while serotonin remained chronically elevated, close to its physiological peak (Figure 1G,H; Supplementary Figure 2A,B).

### 3.2 Circadian Disruption Exacerbates Cisplatin‐Induced Ototoxicity

To further investigate the impact of circadian disruption on cisplatin‐induced ototoxicity, mice maintained in their respective lighting conditions received three cycles of cisplatin treatment (Figure 1A). Prior to chemotherapy, no significant differences were observed in auditory function among the three groups (Figure 1B,C). Following chemotherapy, all groups exhibited elevated thresholds across frequencies in both ABR and DPOAE tests. However, low‐frequency auditory thresholds in the Light–Dark group were significantly more preserved than those in the Light–Light and Dark–Dark groups (Figure 1D,F). We further compared cochlear hair cell counts, auditory nerve fiber bundles, and ribbon synapses among the three groups before and after chemotherapy. Prior to treatment, no significant differences were noted in cochlear morphology or synaptic counts (Figure 1J–N; Supplementary Figure 3A,C). After cisplatin administration, inner hair cell counts did not differ significantly between the Light–Light/Dark–Dark groups and the Light–Dark group. In contrast, the Light–Light and Dark–Dark groups showed significantly greater outer hair cell loss across the apical, middle, and basal turns of the cochlea (Figure 1J′,M). Moreover, although not statistically significant, the Light–Light and Dark–Dark groups exhibited a decreasing trend in the number of peripheral auditory nerve projections and ribbon synapses from the apical to the basal turns (Figure 1J′,N; Supplementary Figure 3B,D).

Given that the most pronounced differences in cochlear and serum melatonin/serotonin levels among the three groups occurred at ZT0 before chemotherapy, we measured these hormone levels in the cochlea and serum at ZT0 on the final day of chemotherapy (Day 42) to assess the effect of chemotherapy on hormonal regulation. Compared with the same zeitgeber time point prior to chemotherapy, all groups showed reduced melatonin levels and elevated serotonin levels in both cochlear tissue and serum. However, the differences in cochlear and serum melatonin/serotonin content between the two circadian‐disrupted groups and the Light–Dark group were markedly smaller after chemotherapy (Figure 1I; Supplementary Figure 3C). These results suggest that circadian disruption does not independently cause significant hearing loss, but rather increases susceptibility to cisplatin‐induced ototoxicity, likely through the combined modulation of melatonin/serotonin balance by both circadian rhythms and cisplatin treatment.

### 3.3 Melatonin/Serotonin Modulates the Prognosis of Cisplatin‐Induced Ototoxicity

**FIGURE 2:**
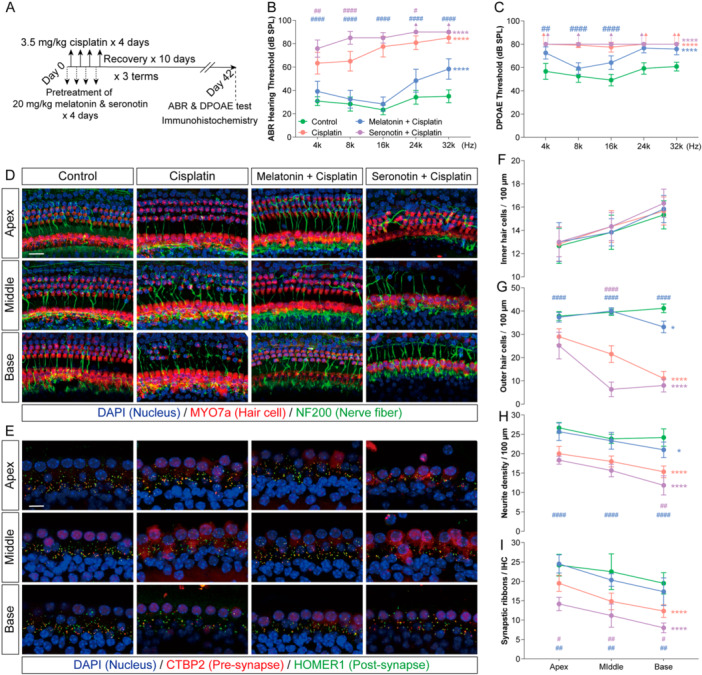
Melatonin and serotonin modulate the prognosis of cisplatin‐induced ototoxicity. (A) Schematic of experimental design combining melatonin or serotonin intervention with cisplatin chemotherapy in mice. (B and C) ABR and DPOAE thresholds after chemotherapy in the indicated groups. (D) Representative immunofluorescence images of the apical, middle, and basal turns of the cochlear basilar membrane after chemotherapy. Myosin 7a (red) labels hair cells, Neurofilament (green) labels auditory nerve fibers, and DAPI (blue) stains nuclei. Scale bars = 20 μm. (E) Representative immunofluorescence images of the ribbon‐synapse region in inner hair cells across cochlear turns after chemotherapy. CtBP2 (red) labels presynaptic ribbons, Homer1 (green) labels postsynaptic densities, and DAPI (blue) stains nuclei. Scale bars = 10 μm. (F–I) Quantitative analysis of inner hair cell density (F), outer hair cell density (G), auditory nerve fiber terminal density (H), and intact ribbon‐synapse density (I) in the apical, middle, and basal cochlear turns after chemotherapy. Data are presented as mean ± SEM. *p < 0.05, **p < 0.01, ***p < 0.001, ****p < 0.0001 vs. Control all frequencies/turns (corrected for multiple comparisons); ^###^ *p* < 0.001, ^####^ *p* < 0.0001 vs. Cisplatin on each frequencies/turns (corrected for multiple comparisons); two‐way ANOVA with Bonferroni post hoc test, *n* = 6.

To investigate the role of melatonin/serotonin regulation in cisplatin‐associated hearing loss, we administered exogenous melatonin or serotonin (20 mg/kg) as a pretreatment prior to cisplatin chemotherapy in mice (Figure 2A) [24]. After chemotherapy, ABR and DPOAE thresholds were elevated across all frequencies, with the most pronounced shifts observed at higher frequencies. We further found that melatonin pretreatment mitigated these threshold elevations, whereas serotonin pretreatment exacerbated them (Figure 2B,C). No significant change in inner hair cell counts was observed among the groups. However, cisplatin‐treated cochleae exhibited marked reductions in outer hair cells, auditory nerve fibers, and ribbon synapses, with the extent of loss increasing from the apical toward the basal turns. While serotonin worsened this damage, melatonin conferred protection, preserving outer hair cell numbers, nerve fibers, and ribbon synapses, and restoring these counts to near‐physiological levels in the apical and middle turns (Figure 2D–I). Together, these findings indicate that melatonin and serotonin differentially regulate cisplatin‐induced ototoxicity: systemic melatonin attenuates cisplatin‐mediated hearing loss and cochlear damage, whereas systemic serotonin aggravates the injury.

**FIGURE 3:**
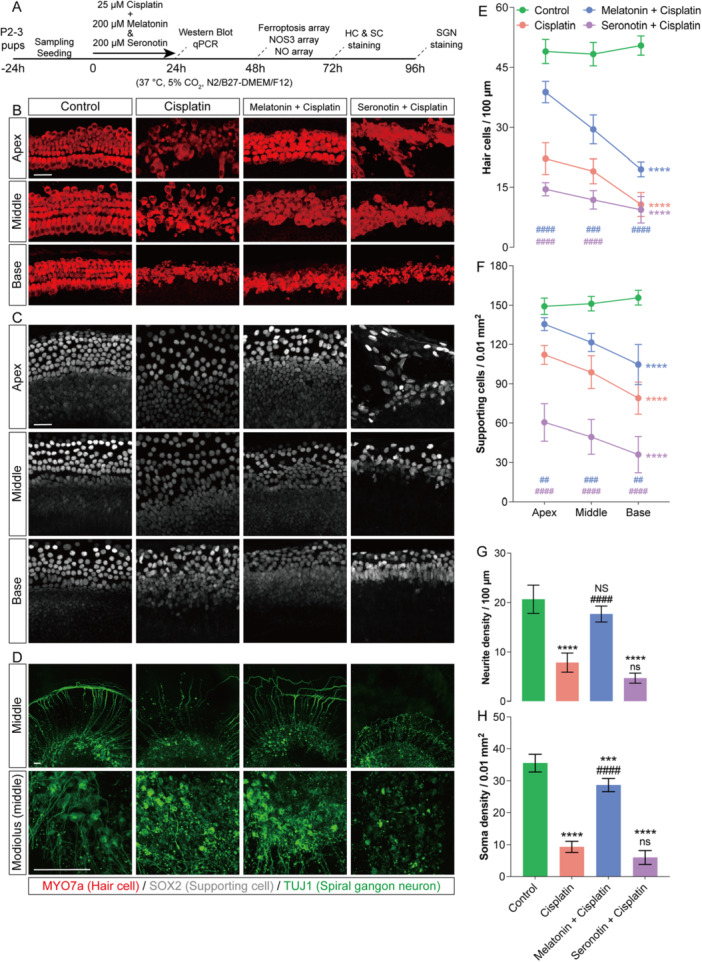
Melatonin and serotonin regulate cell survival in cisplatin‐treated cochlear explants in vitro. (A) Schematic of the experimental workflow: neonatal mouse cochlear explant culture, drug treatment, followed by sampling and seeding. (B–D) Representative immunofluorescence images of apical, middle, and basal turns of cochlear basilar membrane explants and spiral ganglion explants after 72 h of culture in the Control, Cisplatin, Cisplatin + Melatonin, and Cisplatin + Serotonin groups. Myosin 7a (red) labels hair cells, SOX2 (gray) labels supporting cells, and Tuj1 (green) labels spiral ganglion neurons and their neurites. (E–H) Quantitative analysis of hair‐cell and supporting‐cell densities across cochlear turns (E–G), as well as neurite density in the middle turn and neuronal soma density in the modiolar region (H). Data are presented as mean ± SEM. *p < 0.05, **p < 0.01, ***p < 0.001, ****p < 0.0001 vs. Control group (corrected for multiple comparisons); ^###^ *p* < 0.001, ^####^ *p* < 0.0001 vs. Cisplatin group (corrected for multiple comparisons); two‐way ANOVA with Bonferroni post hoc test, *n* = 6, Scale bars = 40 μm.

To further investigate the direct effects of melatonin and serotonin, we employed an in vitro cisplatin‐injured cochlear explant model (Figure 3A). As the ELISA array indicated that serotonin levels in mouse inner‐ear tissues are approximately 10‐fold higher than those of melatonin, the in vitro pretreatment concentrations were set at 100 μM for melatonin and 1 mM for serotonin, based on the methodology described by Zheng et al [20]. In cochlear explants, cisplatin disrupted the organization of hair cells and supporting cells, reduced the number of spiral ganglion neuron fibers and somata, and led to the loss of the outer spiral bundle. Consistent with the in vivo pharmacological observations, serotonin further aggravated cisplatin‐induced cochlear cell death. In contrast, melatonin alleviated cisplatin‐mediated damage to hair cells and supporting cells, restored linear cellular arrangement in the apical and middle turns, and protected spiral ganglion neurons by reducing cell lysis, increasing peripheral projections, and preserving the structure of the outer spiral bundle (Figure 3B–D). These results demonstrate that the balance between melatonin and serotonin directly modulates cisplatin‐induced ototoxicity, with melatonin exerting a protective effect and serotonin exacerbating cochlear injury.

### 3.4 NOS3–NO Pathway Involves the Regulation of Cisplatin‐Induced Ototoxicity by Melatonin and Serotonin

**FIGURE 4:**
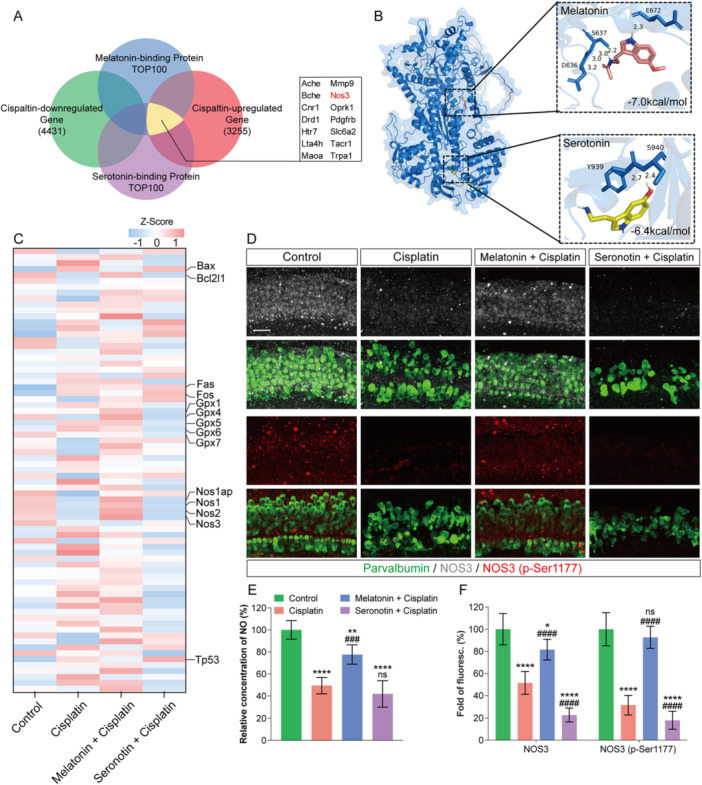
Melatonin and serotonin mediate the regulation of cisplatin‐induced ototoxicity through the NOS3–NO pathway. (A) Venn diagram showing the overlap between differentially expressed genes in cochlear explants after cisplatin injury and high‐affinity target proteins predicted for melatonin and serotonin using SwissTargetPrediction. Nine cisplatin‐downregulated genes, including NOS3, were identified as potential binding targets for both ligands. (B) AutoDock modeling of the high‐affinity binding sites and binding affinities of melatonin (red) and serotonin (yellow) with the NOS3 protein. (C) qPCR analysis of nitric oxide (NO) signaling pathway‐related factors in cochlear explants after 24 h of culture in the indicated groups. Data are shown as Z‐scores. (D) Representative immunofluorescence images of the middle turn of cochlear basilar membrane explants cultured for 48 h. Parvalbumin (green) labels hair cells, showing colocalization with NOS3 (red) and active (phosphorylated) NOS3 (gray). Scale bars = 30 μm. (E) Tissue NO content in cochlear explants after 48 h of culture in the indicated groups. (F) Quantitative analysis of the mean fluorescence intensity of NOS3 and active (phosphorylated) NOS3 within the hair‐cell region from panel (D). Data are presented as mean ± SEM. *p < 0.05, **p < 0.01, ***p < 0.001, ****p < 0.0001 vs. Control group; ^###^ *p* < 0.001, ^####^ *p* < 0.0001 vs. Cisplatin group; two‐way ANOVA with Bonferroni post hoc test, *n* = 6.

Given the structural similarity between melatonin and serotonin, we hypothesized that their binding protein profiles would exhibit substantial overlap. By integrating transcriptomic data from cisplatin‐damaged cochlear explants with SwissTargetPrediction analysis, we screened for proteins that were downregulated by cisplatin and were predicted to bind both ligands, identifying nine candidate proteins (Figure 4A). Among these, NOS3, known to be involved in nitric oxide (NO) synthesis and sensorineural hearing loss, has not been elucidated in the context of cisplatin‐induced ototoxicity. Analysis of NO‐pathway‐related gene expression in cochlear explants revealed that cisplatin downregulated genes of the NOS family. Co‐treatment with melatonin reversed cisplatin‐mediated alterations in NO biosynthesis, superoxide metabolism, and oxidative‐stress‐related genes, whereas co‐treatment with serotonin exacerbated the expression of stress‐ and damage‐associated genes (Figure 4C). These findings implicate that melatonin and serotonin may modulate cisplatin‐induced ototoxicity via the NOS3–NO pathway. To preliminarily examine the antagonistic effects of melatonin and serotonin on NOS3, we employed AutoDock to simulate their binding kinetics. Molecular docking demonstrated that melatonin preferentially binds to NOS3 residues 636, 637, and 672, while serotonin favors interactions with residues 939 and 949, indicating distinct binding patterns between the two ligands (Figure 4B).

We next assessed NOS3 expression in cochlear tissues via immunofluorescence analysis. In the organ of Corti, cisplatin treatment downregulated total NOS3 protein levels and reduced phosphorylation at Ser1177, a posttranslational modification essential for its catalytic activity. Co‐administration of melatonin restored both NOS3 expression and Ser1177 phosphorylation, while serotonin further suppressed NOS3 and correspondingly diminished its phosphorylation (Figure 4D,F). Cisplatin also attenuated nitric oxide (NO) production—an effect that was rescued by melatonin but exacerbated by serotonin co‐treatment (Figure 4E). To functionally validate the protective role of NOS3, we supplemented cisplatin‐exposed cochlear explants with recombinant NOS3 protein. This intervention significantly promoted the survival of hair cells (HCs), supporting cells, and spiral ganglion neurons (SGNs) following cisplatin injury (Supplementary Figure 4). Collectively, these data demonstrate that cisplatin‐induced ototoxicity involves suppression of the NOS3–NO axis, which is differentially modulated by melatonin (restorative) and serotonin (exacerbative).

### 3.5 NOS3–NO Pathway Protects Hair Cells From Cisplatin‐Induced Ferroptosis by Activating CREB

Quantitative PCR analysis revealed distinct expression profiles of GPX family genes across treatment groups. Notably, GPX4 expression responded to cisplatin, melatonin, and serotonin in a pattern similar to that of NOS3 (Figure 4C). Given the central role of GPX4 in detoxifying lipid peroxides and inhibiting ferroptosis, we examined whether melatonin and serotonin influence this form of regulated cell death. Consistent with previous reports, cisplatin elevated fluorescence intensities of MitoSOX, BODIPY, FerroOrange, and MitoFerroGreen in hair cells, indicating increased mitochondrial superoxide, lipid peroxidation, labile iron, and mitochondrial iron, respectively (Supplementary Figure 5A–F). Co‐treatment with melatonin attenuated these signals, whereas serotonin exacerbated them, suggesting that melatonin and serotonin oppositely modulate cisplatin‐induced oxidative stress, lipid peroxidation, and iron dysregulation. Western blotting further demonstrated that cisplatin up‐regulated 4‐hydroxynonenal (4‐HNE) and lipid peroxide (LPO) levels, while down‐regulating GPX4 and SLC7A11. These changes were reversed by melatonin but aggravated by serotonin (Supplementary Figure 5G–L). Together, these data indicate that melatonin and serotonin differentially regulate cisplatin‐triggered ferroptosis in the cochlea, thereby influencing susceptibility to ototoxicity.

The regulatory effects of melatonin and serotonin indicated that their downstream target, NOS3, likely mediates the protection against cisplatin‐induced dysregulation of iron metabolism. To test this, we assessed intracellular iron levels and oxidative stress in cochlear explants co‐treated with recombinant NOS3 and cisplatin. Increasing NOS3 activity attenuated the cisplatin‐induced elevation in fluorescence signals of MitoSOX, BODIPY, FerroOrange, and MitoFerroGreen (Supplementary Figure 6A–F), and suppressed cisplatin‐triggered phosphorylation of p53. Furthermore, NOS3 overexpression significantly reduced the upregulation of 4‐hydroxynonenal (4‐HNE), lipid peroxides (LPO), and ferritin heavy chain (FTH1), while restoring the expression of SLC7A11 (Supplementary Figure 6G–M). These results demonstrate that NOS3 inhibits the p53 signaling pathway, reduces lipid peroxide accumulation, alleviates iron overload, and consequently attenuates ferroptosis.

**FIGURE 5:**
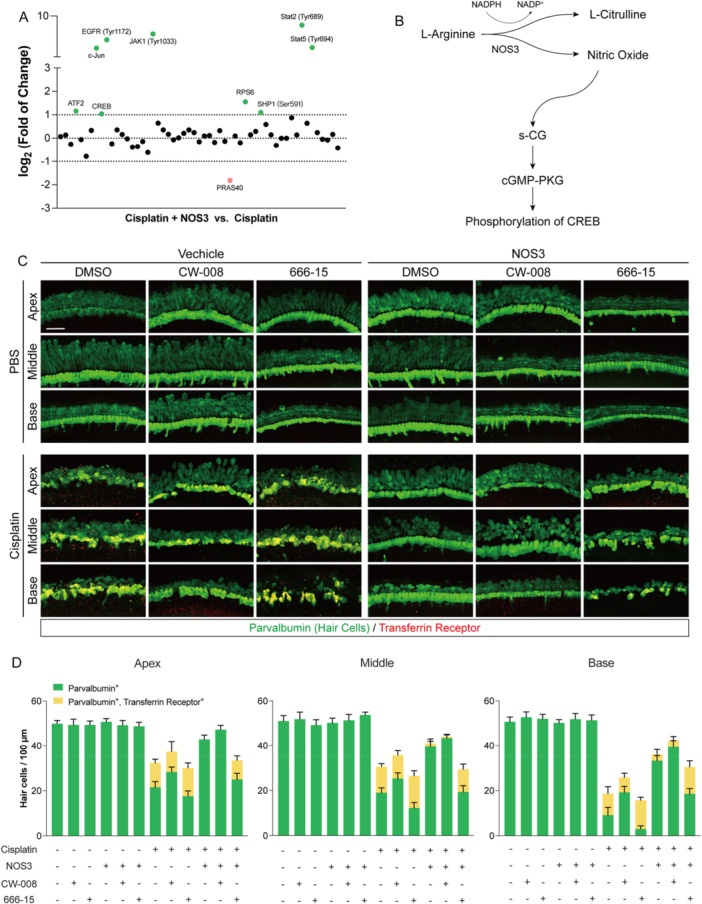
CREB acts downstream of the melatonin/serotonin and NOS3–NO pathways to suppress cisplatin‐induced ferroptosis. (A) Heatmap showing the phosphorylation levels of 55 proteins in cochlear explants comparing the NOS3 + Cisplatin group vs. the Cisplatin‐alone group (green: higher phosphorylation in NOS3 + Cisplatin; red: lower phosphorylation). (B) Schematic diagram of the NOS3/NO/cGMP/PKG signaling cascade. (C) Representative immunofluorescence images of cochlear explants after 48 h of treatment with cisplatin, NOS3 agonist, CW‐008, or 666‐15. Parvalbumin (green) labels hair cells; transferrin receptor (red) marks ferroptotic cells. Scale bars = 40 μm. (D) Quantitative analysis of Parvalbumin‐positive hair cells and Parvalbumin/transferrin receptor double‐positive ferroptotic cells based on the images in panel (C). Data are presented as mean ± SEM, *n* = 6.

While the anti‐ferroptotic effect of the NOS3–NO axis has been established, its underlying mechanism, whether it acts directly through the reductive activity of NO or requires additional signaling mediators, remains unclear. To address this, we performed a comparative phospho‐proteomic analysis of basilar membrane tissues from cisplatin‐only and NOS3+cisplatin‐treated groups. NOS3 co‐treatment significantly elevated the phosphorylation levels of multiple proteins, including ATF2, CREB, c‐Jun, EGFR, JAK1, RPS6, SHP‐1, STAT2, and STAT5, in cisplatin‐injured tissues (Figure 5A). Among these, CREB—a transcription factor activated by cAMP binding that regulates neuronal function and plasticity—was of particular interest [29]. Previous studies indicate that NO‐activated cGMP‐PKG signaling can phosphorylate CREB at Ser^133^ to enhance its transcriptional activity (Figure 5B) [30]. To examine the functional role of CREB in cisplatin‐induced ferroptosis, we pharmacologically modulated CREB activity using the agonist CW‐008 and the inhibitor 666‐15. Neither CW‐008, 666‐15, nor NOS3 alone affected hair cell counts or induced TfR‐positive cells in control explants. In cisplatin‐damaged explants, however, CW‐008 increased hair cell survival and suppressed TfR expression, whereas 666‐15 exacerbated hair cell loss and raised the proportion of TfR‐positive hair cells. We next asked whether CREB activity influences the protective effect of NOS3. Compared with the NOS3+cisplatin group, adding CW‐008 further increased hair cell survival across all cochlear turns and markedly reduced TfR‐positive cells in the middle turn. In contrast, 666‐15 diminished hair cell numbers and increased TfR positivity from the apical to basal turns (Figure 5C,D). Together, these results indicate that CREB activation synergizes with NOS3‐mediated protection, and that the NOS3–NO pathway suppresses cisplatin‐induced hair cell ferroptosis in a manner dependent on CREB phosphorylation.

### 3.6 NOS3 Rescues Cochlear Cells From Cisplatin‐Induced Ototoxicity In Vivo

**FIGURE 6:**
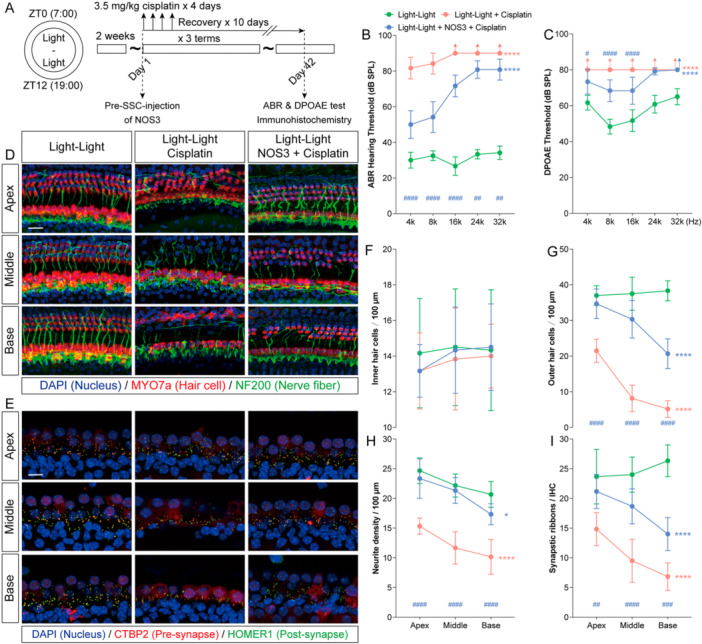
NOS3 reduces cisplatin‐induced ototoxicity in circadian rhythm‐disrupted mice. (A) Schematic diagram of the experimental design combining cisplatin chemotherapy with NOS3 treatment in mice with disrupted circadian rhythms. (B and C) Post‐treatment auditory brainstem response (ABR) and distortion‐product otoacoustic emission (DPOAE) thresholds in the Control, Cisplatin, and Cisplatin + NOS3 groups. (D) Representative immunofluorescence images of the apical, middle, and basal turns of the cochlea after treatment, stained for Myosin 7a (red, hair cells), Neurofilament (green, nerve fibers), and DAPI (blue, nuclei). Scale bars = 20 μm. (E) Representative immunofluorescence images of inner hair cell ribbon‐synapse regions in the three cochlear turns, labeled for Ctbp2 (red, presynaptic ribbons), Homer1 (green, postsynaptic densities), and DAPI (blue, nuclei). Scale bars = 10 μm. (F–I) Quantitative analysis of inner hair cells, outer hair cells, auditory nerve‐fiber terminals, and intact ribbon‐synapse density in the apical, middle, and basal turns after treatment. Data are presented as mean ± SEM. *p < 0.05, **p < 0.01, ***p < 0.001, ****p < 0.0001 vs. Control (Light–Dark) group on all frequencies/turns (corrected for multiple comparisons); two‐way ANOVA with Bonferroni post hoc test, *n* = 6.

Given the selective permeability of the blood–labyrinth barrier, we evaluated the therapeutic potential of NOS3 in vivo via semicircular canal injection. To minimize potential confounding effects of endogenous melatonin and serotonin on NOS3 activity, experiments were performed using a Light–Light model, which displays pronounced circadian disruption (Figure 6A). Prophylactic administration of recombinant NOS3 protein before chemotherapy significantly reversed the cisplatin‐induced elevation in auditory brainstem response thresholds across all tested frequencies, with particularly notable recovery of low‐frequency auditory function and distortion‐product otoacoustic emission responses (Figure 6B,C). Moreover, NOS3 treatment markedly restored the numbers of outer hair cells, auditory nerve fiber bundles, and ribbon synapses (Figure 6D–I).

We further investigated whether NOS3 retains its protective efficacy under normal chemotherapeutic conditions in mice maintained on a light–dark cycle (Supplementary Figure 7A). In this model, NOS3 administration confined hearing loss to a mild range across all frequencies and significantly reduced distortion‐product otoacoustic emission thresholds (Supplementary Figure 7B,C). Moreover, NOS3 effectively rescued outer hair cells in the apical, middle, and basal turns of the cochlea, prevented degeneration of auditory nerve fibers, and preserved synaptic morphology and density. In the NOS3 + cisplatin group, the counts of hair cells and nerve fibers in the apical and middle turns showed no significant difference compared with the control group (Supplementary Figure 7D–I).

**FIGURE 7:**
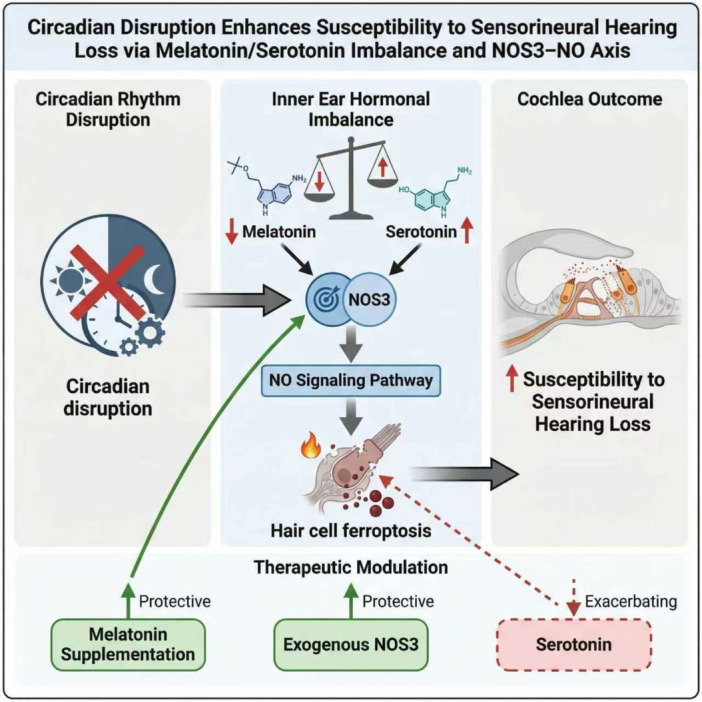
Circadian‐related Serotonin/Melatonin level Modulates Cisplatin Ototoxicity Susceptibility depended on NOS3–NO pathway. Circadian disruption upsets melatonin/serotonin balance in the inner ear. Melatonin protects against cisplatin‐induced ototoxicity, whereas serotonin worsens damage. This opposing regulation acts through NOS3 to modulate NO signaling and ferroptosis. Consequently, the melatonin/serotonin balance determines susceptibility to sensorineural hearing loss, revealing a mechanistic link between lifestyle‐related hormone dysregulation and cochlear vulnerability.

These findings further demonstrate that NOS3 acts as a downstream mediator through which the melatonin/serotonin balance regulates susceptibility to cisplatin‐induced ototoxicity, underscoring its potential as a therapeutic target for alleviating cisplatin‐related hearing loss (Figure 7).

## 4 Discussion

Studies in animal models and human cohorts have shown that sleep disturbances and circadian rhythm disruptions increase susceptibility to sensorineural hearing loss. Our research further reveals that individual circadian rhythm disorders exacerbate sensitivity to cisplatin‐induced ototoxicity and alter the levels of melatonin and serotonin in inner ear tissues. Through combination therapy experiments, we have confirmed that these two hormones can regulate the injury process of inner ear cells. Moreover, melatonin, which exerts protective effects, also exhibits anti‐tumor activity [31, 32]. Molecular interaction prediction analysis indicates that the NOS3–NO pathway may be involved in cisplatin‐mediated oxidative stress and iron overload, thereby regulating ferroptosis in inner ear cells. This process ultimately affects the survival and synaptic function of hair cells and spiral ganglion neurons. These findings highlight the critical role of the circadian clock in auditory protection, as well as the important functions of endogenous hormones in maintaining inner ear homeostasis and resisting damage.

Cisplatin chemotherapy influences humoral regulation, for example by inducing serotonin release from peripheral blood mononuclear cells [33]. In patients with non‐small cell lung cancer, melatonin levels are already reduced and decrease further after cisplatin‐based chemotherapy [34]. While its systemic effects are documented, the specific impact of cisplatin on cochlear metabolism remained unclear. This study found that cisplatin‐induced side effects are associated with individual circadian physiology and alter inner ear melatonin and serotonin levels. Circadian disruption reduces melatonin and elevates serotonin locally. Prolonged cisplatin treatment further disrupts the synthesis and release of these hormones, increasing inner ear cell sensitivity to chemotherapy. Exogenous melatonin supplementation effectively counteracts cisplatin ototoxicity, whereas serotonin exacerbates the damage. This suggests that targeted modulation of the melatonin/serotonin balance could enhance inner ear resistance and mitigate hearing impairment.

Both serotonin and melatonin, as structurally related indole compounds, bind to specific G‐protein‐coupled receptors (serotonin and melatonin receptors) and classically activate the cAMP signaling pathway to modulate central and peripheral functions [35, 36, 37]. However, in cisplatin‐injured cochlear explants, no significant transcriptional alterations in these receptors were detected. This indicates that melatonin and serotonin may regulate cisplatin‐induced inner‐ear damage through non‐canonical, receptor‐independent mechanisms. Further integrated screening of cisplatin‐induced differentially expressed genes and serotonin/melatonin‐interacting proteins identified NOS3 as a potential therapeutic target for cisplatin‐induced ototoxicity. Although both hormones can interact with NOS3, they exhibit differential binding preferences: melatonin displays higher affinity for the N‐terminal domain, whereas serotonin binds more preferentially to the C‐terminal region. Previous studies have established that NOS3 activity is predominantly regulated via phosphorylation by upstream kinases. Ser1177, located within the catalytic domain, represents a critical phosphorylation site whose modification markedly enhances enzymatic activity [38, 39]. In the present study, we demonstrate that melatonin sustains NOS3 expression and promotes phosphorylation at its active site, whereas serotonin exacerbates cisplatin‐mediated downregulation of NOS3 and induces dephosphorylation of the same site. This differential effect may be attributable to the closer proximity of serotonin binding to the catalytic center, which could induce conformational changes in NOS3, thereby inhibiting activating phosphorylation and reducing protein stability.

Clinical and genome‐wide analyses have shown that polymorphisms in the NOS3 gene are closely associated with the incidence of idiopathic sudden sensorineural hearing loss and Meniere's disease [40, 41, 42, 43]. Moreover, decreased NOS3 activity in the murine inner ear leads to the development of sensorineural hearing loss [44, 45]. However, the mechanism by which NOS3 protects against cisplatin‐mediated hair‐cell damage remains unclear. As a calcium‐dependent enzyme, NOS3 catalyzes the conversion of l‐arginine to nitric oxide (NO) and l‐citrulline. NO acts as a key signaling molecule that facilitates intercellular communication, regulates cardiovascular tone, modulates immune responses, and participates in membrane metabolism [46, 47, 48, 49]. In the mammalian cochlea, NO functions as a vasodilator and neuromodulator, and moderately elevated NO levels are often correlated with enhanced resistance to injury [50, 51, 52, 53, 54, 55, 56]. Nevertheless, direct evidence regarding the protective role of NO against cisplatin‐related ototoxicity is still limited.

By investigating the regulatory effects of melatonin/serotonin on the cisplatin‐suppressed NO pathway, we observed that melatonin effectively restored the transcriptional level of GPX4, whereas serotonin further inhibited its expression. Among glutathione peroxidase isoforms, GPX4 is uniquely capable of reducing esterified oxidized fatty acids and cholesterol hydroperoxides, and it serves as a critical factor in protecting inner‐ear cells against cisplatin‐induced ferroptosis [57, 58, 59]. Our experimental data further indicate that activation of the NOS3–NO pathway suppresses oxidative stress and lipid peroxidation in hair cells, alleviates intracellular iron overload, upregulates anti‐ferroptotic genes, and downregulates pro‐ferroptotic genes. Collectively, these findings suggest that the NOS3–NO pathway protects hair cells by modulating cisplatin‐triggered ferroptosis.

Nitric oxide (NO) possesses reducing properties and can react with superoxide anions or other free radicals to form species such as nitroxyl (HNO/NO^−^) or nitrogen dioxide (NO_2_). However, in mammals, the absence of efficient nitro‐metabolizing enzymes leads to the conversion of NO into reactive nitrogen species (RNS), which can exacerbate oxidative stress [60]. Under physiological conditions, NO binds to the heme within the Heme‐NO/Oxygen binding (H‐NOX) domain of the soluble guanylate cyclase (sGC) β subunit. This binding induces a conformational rotation in the enzyme's cyclase domain, which opens the GTP‐binding site and activates the synthesis of cyclic guanosine monophosphate (cGMP) [61]. Protein kinase G (PKG) is activated upon cGMP binding, which releases its catalytic domain from autoinhibition. This allows PKG to phosphorylate downstream targets, many of which overlap with substrates of protein kinase A (PKA) [62, 63]. The cGMP/PKG pathway protects against hearing loss through several distinct mechanisms: sGC helps preserve the integrity of inner hair cell ribbon synapses; natriuretic peptide receptor A (GC‐A) enhances the auditory system's resistance to stress; and natriuretic peptide receptor B (GC‐B) is involved in the central modulation of auditory signaling [64]. Consistent with this protective role, mice deficient in the *Prkg1* gene (encoding PKG) show increased susceptibility to acoustic trauma. Conversely, inhibition of phosphodiesterase 5 (PDE5) by vardenafil prevents cGMP hydrolysis, activates Poly(ADP‐ribose) polymerase (PARP), and confers protection against noise‐induced hearing loss [65]. In vitro, the cGMP/PKG pathway exerts neurotrophic and protective effects, promoting neuronal survival and axonal extension while inhibiting apoptosis [66, 67]. Furthermore, the NOS/NO/cGMP/PKG signaling cascade is calcium‐dependent and functions as a calcium‐sensing checkpoint, providing negative feedback to regulate calcium homeostasis in hair cells [68, 69, 70]. In our phosphoproteomic analysis of cochlear explants, CREB emerged as the sole downstream target of PKG whose phosphorylation was specifically induced by NOS3. Previous studies have established that CREB mitigates noise‐ or ototoxin‐induced hearing loss, promotes axonal regeneration and synaptic function, and suppresses apoptosis. Here, by employing both pharmacological agonists and inhibitors of CREB, we demonstrate that NOS3 attenuates cisplatin‐induced hair cell ferroptosis in a CREB‐dependent manner, which aligns with the previously reported protective roles of CREB [50, 71, 72, 73].

Pisani et al. have proposed that the BDNF/TrkB pathway mediates protection against cisplatin ototoxicity via CREB activation—a mechanism also implicated in the circadian susceptibility to noise‐induced hearing loss [3, 73]. Light exposure resets circadian gene expression by inducing CREB phosphorylation, which facilitates its binding to cAMP response elements (CREs) within core clock genes in the suprachiasmatic nucleus (SCN) [74]. In peripheral cells, circadian rhythmicity is maintained by endoplasmic reticulum (ER) calcium dynamics, regulated through IP3R, CaMK II, and MAPK signaling, and relies on CREB to sustain oscillatory expression of clock genes [75, 76]. Notably, this ER regulatory network is known to be modulated by the PKG pathway. In line with these observations, our previous work identified ER stress as a key pathophysiological process in cisplatin‐induced hair cell ferroptosis [77]. Collectively, this evidence supports the hypothesis that impairment of the NOS3/NO pathway, combined with circadian disruption, heightens susceptibility to cisplatin ototoxicity. Using a Light–Light mouse model subjected to cisplatin chemotherapy, we confirmed that NOS3 administration restores hearing function in mice with circadian disruption. Furthermore, we observed that NOS3 retains its otoprotective efficacy even under normal circadian conditions. This effect may be attributable to the inherent disruptive impact of chemotherapy on the circadian system, whereby exogenous NOS3 supplementation could partially mitigate such dysregulation.

### 4.1 Limitations and Future Perspectives

This study has several inherent limitations, which correspondingly outline important avenues for future research. Further studies are needed to explore other protective pathways downstream of NOS3. For instance, FOXG1 promotes aging hair cell survival via autophagy, while PARP inhibition rescues hearing in Cx26‐null mice through immunoregulation. These findings, together with our demonstration of ferroptosis modulation, suggest that diverse cell death and survival pathways may converge on hair cell integrity. Whether crosstalk exists among these pathways in cisplatin ototoxicity requires future investigation [78, 79]. Additionally, the precise mechanisms underlying cisplatin‐induced perturbations in inner ear melatonin and serotonin homeostasis remain incompletely understood. Prior evidence indicates that the expression of key biosynthetic enzymes (e.g., AANAT, HIOMT, and TPH) is influenced by factors such as age and ototoxic exposure [24, 80]. Future work should therefore aim to characterize the specific effects of cisplatin on these enzymatic and metabolic pathways. A second key limitation is the current absence of selective pharmacological agonists for NOS3. Its activity is known to be regulated indirectly through upstream signaling nodes, including Piezo1/Gq, calcium, AKT, and PKN2 [38, 39]. Consequently, targeting these regulatory pathways represents a rational alternative strategy for therapeutic modulation.

Looking forward, the advent of novel tools such as the “NOckout” fluorescent probe, which enables real‐time, spatially resolved mapping of endogenous NOS3 activity in live cells [81], provides a transformative opportunity. Integrating this technology with high‐throughput small‐molecule screening platforms holds considerable promise for identifying safe and effective NOS3‐targeting compounds for the treatment of inner ear disorders without enhancing tumor neogenesis [82, 83].

## Author Contributions

Siyu Qiu and Shiyi Cai performed the experiments, analyzed the data, and wrote the manuscript. Rong Xie, Chang Liu, and Yingzi He supervised the study and edited the manuscript.

## Conflicts of Interest

The authors declare no conflicts of interest.

## Supporting information

Supporting File 1

Supporting File 2

Supporting File 3

Supporting File 4

Supporting File 5

Supporting File 6

## Data Availability

The data that support the findings of this study are available in the Supplementary lnformation of this article.
