# Supplementary material for: Circadian‐Related Serotonin/Melatonin Level Modulates Cisplatin Ototoxicity Susceptibility Depended on NOS3–NO Pathway
Source: J Pineal Res. 2026 Jun 9;78(4):e70154. doi: 10.1111/jpi.70154 (PMC13248880; doi:10.1111/jpi.70154)
Supplement: Supplementary file 1 — Supporting File 1 [file JPI-78-e70154-s001.docx]

**
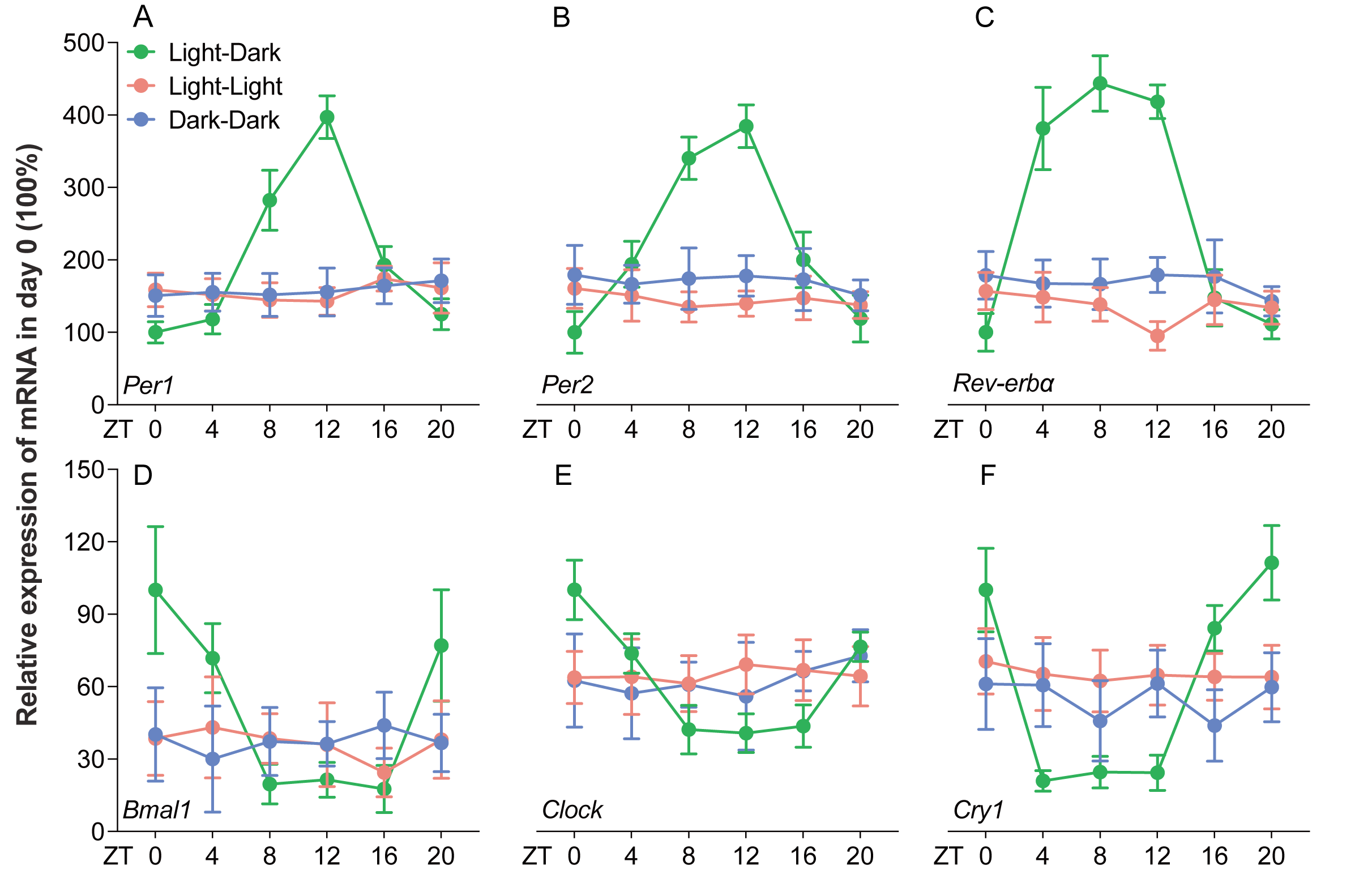
**

**Supplementary Figure 1. Alterations in environmental light induce circadian disruption in mice.**

After two weeks of acclimation under the indicated light conditions, the relative expression levels of circadian clock genes Per1 **(A)**, Per2 **(B)**, Rev‑erbα **(C)**, Bmal1 **(D)**, Clock **(E)**, and Cry1 (F) in cochlear tissues were evaluated by quantitative PCR (qPCR). Expression levels at each time point were normalized to the corresponding value of the Light‑Dark group at Zeitgeber time 0 (ZT0). Data are presented as mean ± SEM. ^*^p < 0.05, ^**^p < 0.01, ^***^p < 0.001, ^****^p < 0.0001 vs. Light‑Dark group; one‑way ANOVA with Bonferroni post‑hoc test, n = 6.


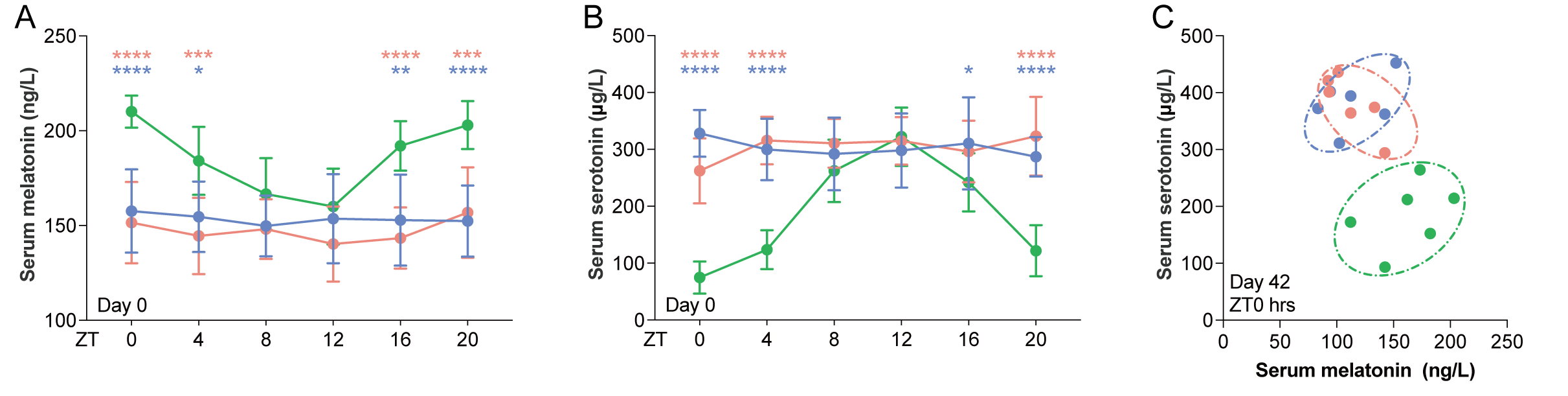


**Supplementary Figure 2. Circadian disruption alters serum melatonin and serotonin oscillations.**

**(A–B)** Circadian profiles of serum melatonin (A) and serotonin (B) concentrations measured at six Zeitgeber time points on the baseline day (Day 0). **(C)** Serum melatonin and serotonin levels at ZT0 on Day 42 after chemotherapy. Data are presented as mean ± SEM. ^*^p < 0.05, ^**^p < 0.01, ^***^p < 0.001, ^****^p < 0.0001 vs. Light‑Dark group; one‑way ANOVA with Bonferroni post‑hoc test, n = 6.


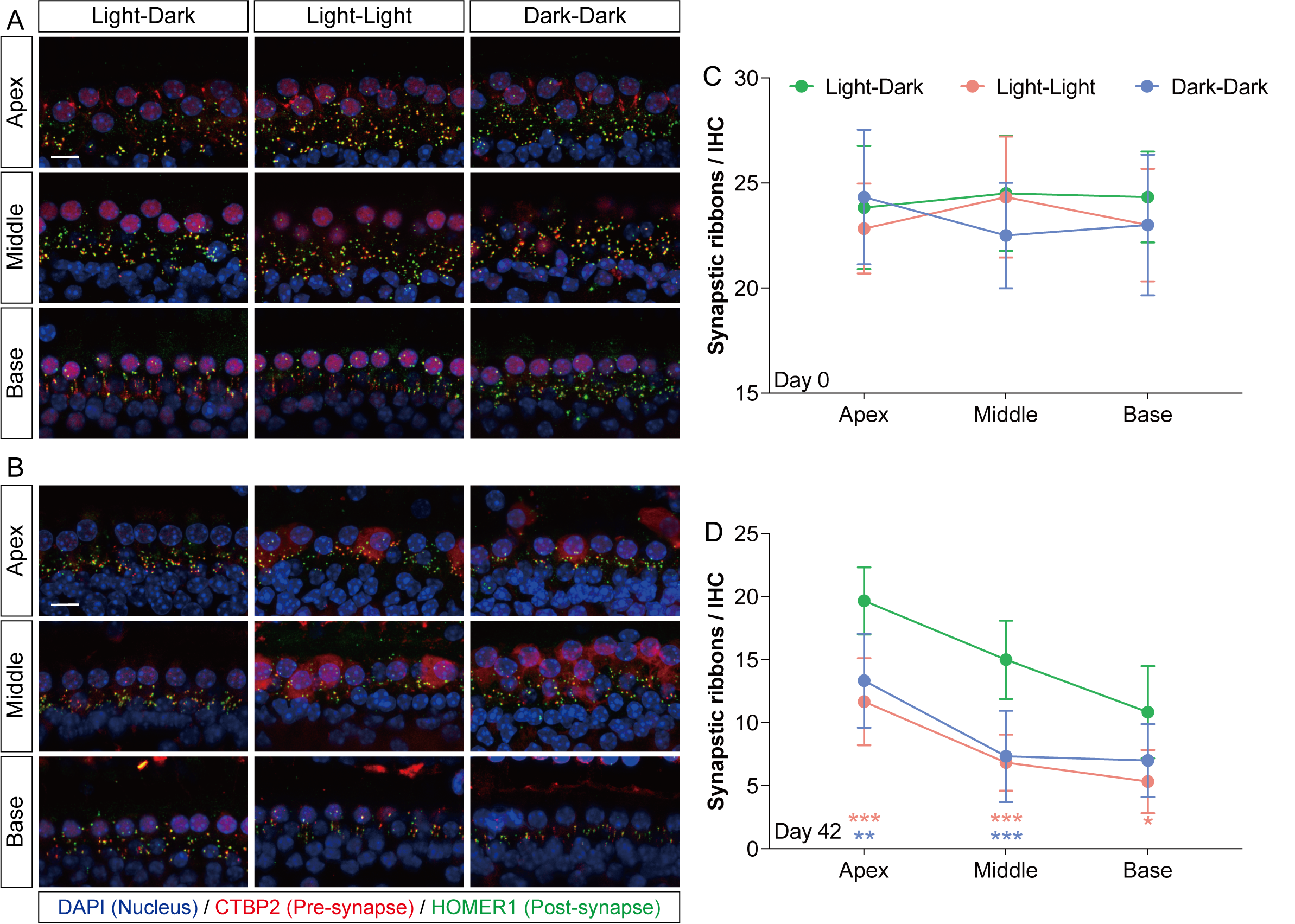


**Supplementary Figure 3. Circadian disruption enhances ribbon‑synapse susceptibility to cisplatin‑induced damage.**

**(A, B)** Representative immunofluorescence images of the ribbon‑synapse region in inner hair cells in the apical, middle, and basal cochlear turns on Day 0 (A) and Day 42 (B). Ctbp2 (red) marks presynaptic ribbons, Homer1 (green) labels postsynaptic densities, and DAPI (blue) stains nuclei. Scale bars = 20 μm. **(C, D)** Quantification of ribbon‑synapse density in outer hair cells in the apical, middle, and basal turns on Day 0 (C) and Day 42 (D). Data are presented as mean ± SEM. ^*^p < 0.05, ^**^p < 0.01, ^***^p < 0.001, ^****^p < 0.0001 vs. Light‑Dark group; one‑way ANOVA with Bonferroni post‑hoc test, n = 6.


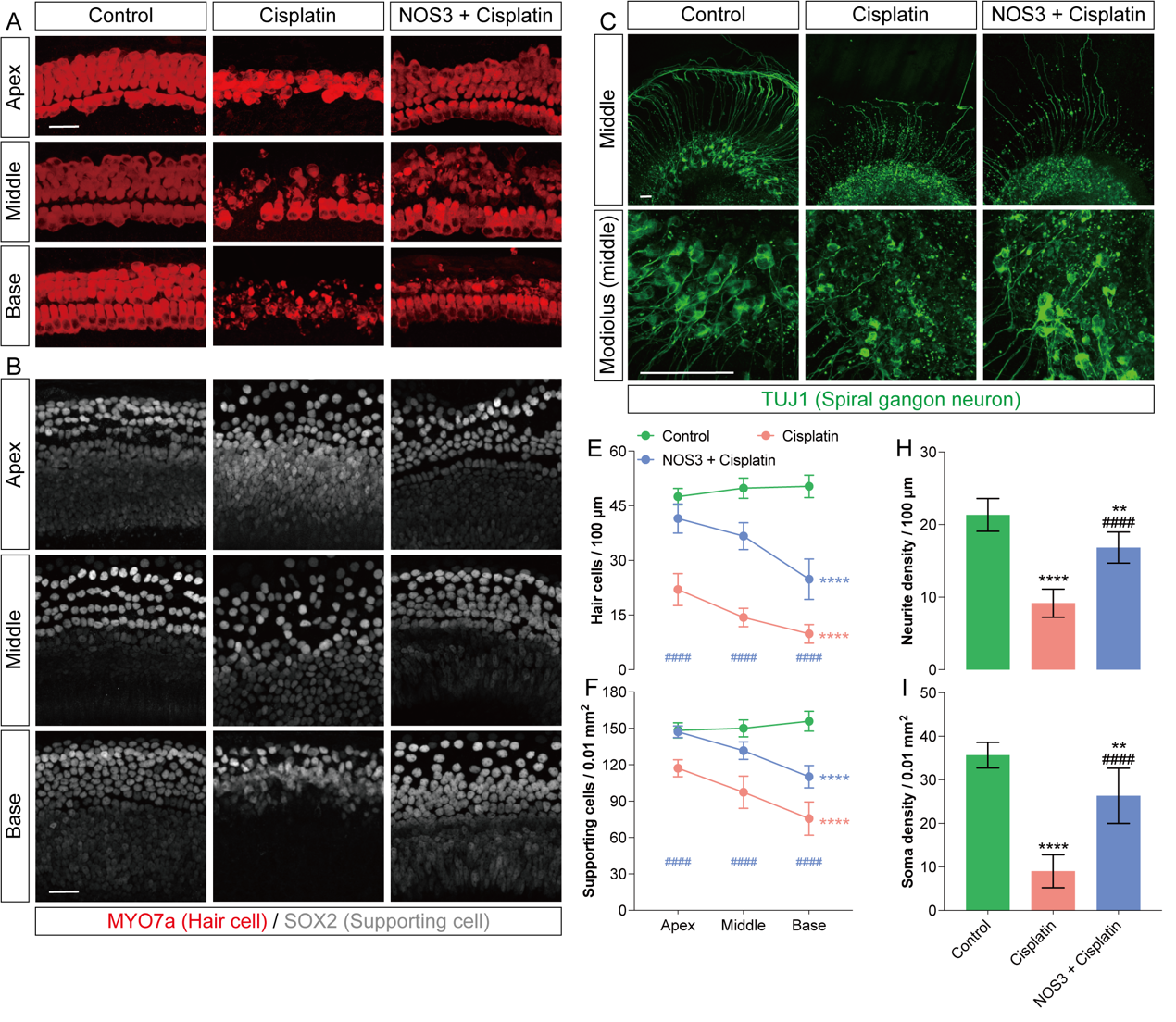


**Supplementary Figure 4. NOS3 reduces cisplatin‑induced cochlear cell death in vitro.**

**(A–C)** Representative immunofluorescence images of the apical, middle, and basal turns of cultured cochlear basilar membrane explants and spiral ganglion explants after 72 h in Control, Cisplatin, and Cisplatin + NOS3 groups. Myosin VIIa (red) labels hair cells, SOX2 (gray) labels supporting cells, and Tuj1 (green) labels spiral ganglion neurons and their neurites. **(D–H)** Quantification of hair‑cell and supporting‑cell density across cochlear turns, together with neurite density in the middle turn and neuronal soma density in the modiolar region. Data are presented as mean ± SEM. ^*^p < 0.05, ^**^p < 0.01, ^***^p < 0.001, ^****^p < 0.0001 vs. Control group; one‑way ANOVA with Bonferroni post‑hoc test, n = 6, Scale bars = 40 μm.


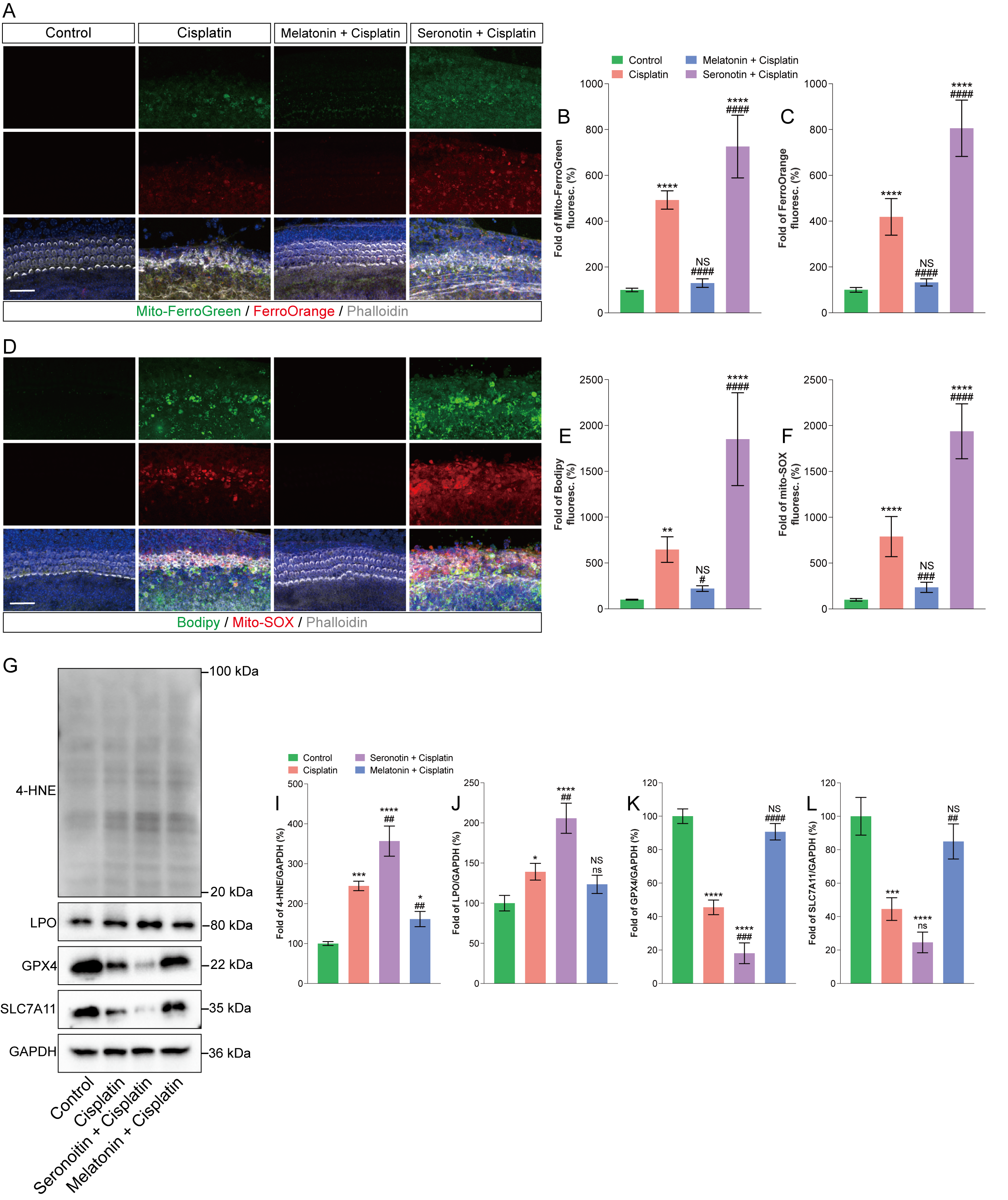


**Supplementary Figure 5. Melatonin and serotonin mitigate cisplatin ototoxicity via ferroptosis.**

**(A)** Representative immunofluorescence images of the middle turn in cultured cochlear basilar membrane explants after 48 h of treatment in Control, Cisplatin, Cisplatin + Melatonin, and Cisplatin + Serotonin groups. Hair cells (labeled with phalloidin, gray) are colocalized with Mito-FerroGreen (green; mitochondrial labile iron) and FerroOrange (red; cytosolic labile iron). Scale bars = 30 μm. **(B, C)** Quantification of mean fluorescence intensity for Mito-FerroGreen (B) and FerroOrange (C) within the hair‑cell region shown in (A). **(D)** Representative immunofluorescence images of the middle turn in explants cultured for 48 h under the indicated treatments. Hair cells (phalloidin, gray) are colocalized with BODIPY 581/591 (green; lipid peroxidation) and MitoSOX Red (red; mitochondrial superoxide). Scale bars = 30 μm. **(E, F)** Quantification of mean fluorescence intensity for BODIPY (E) and MitoSOX Red (F) within the hair‑cell region shown in (D). **(G)** Western blot images of 4‑HNE, LPO, GPX4 and SLC7A11 in cochlear explants cultured for 24 h under the indicated treatments. GAPDH served as loading control. **(H–K)** Quantification of relative protein expression for 4‑HNE (H), LPO (I), GPX4 (J) and SLC7A11 (K), normalized to the Control group. Data are presented as mean ± SEM. ^*^p < 0.05, ^**^p < 0.01, ^***^p < 0.001, ^****^p < 0.0001 vs. Control group; ^####^p < 0.0001, ^###^p < 0.001 vs. Cisplatin group; two‑way ANOVA with Bonferroni post‑hoc test, n = 6.


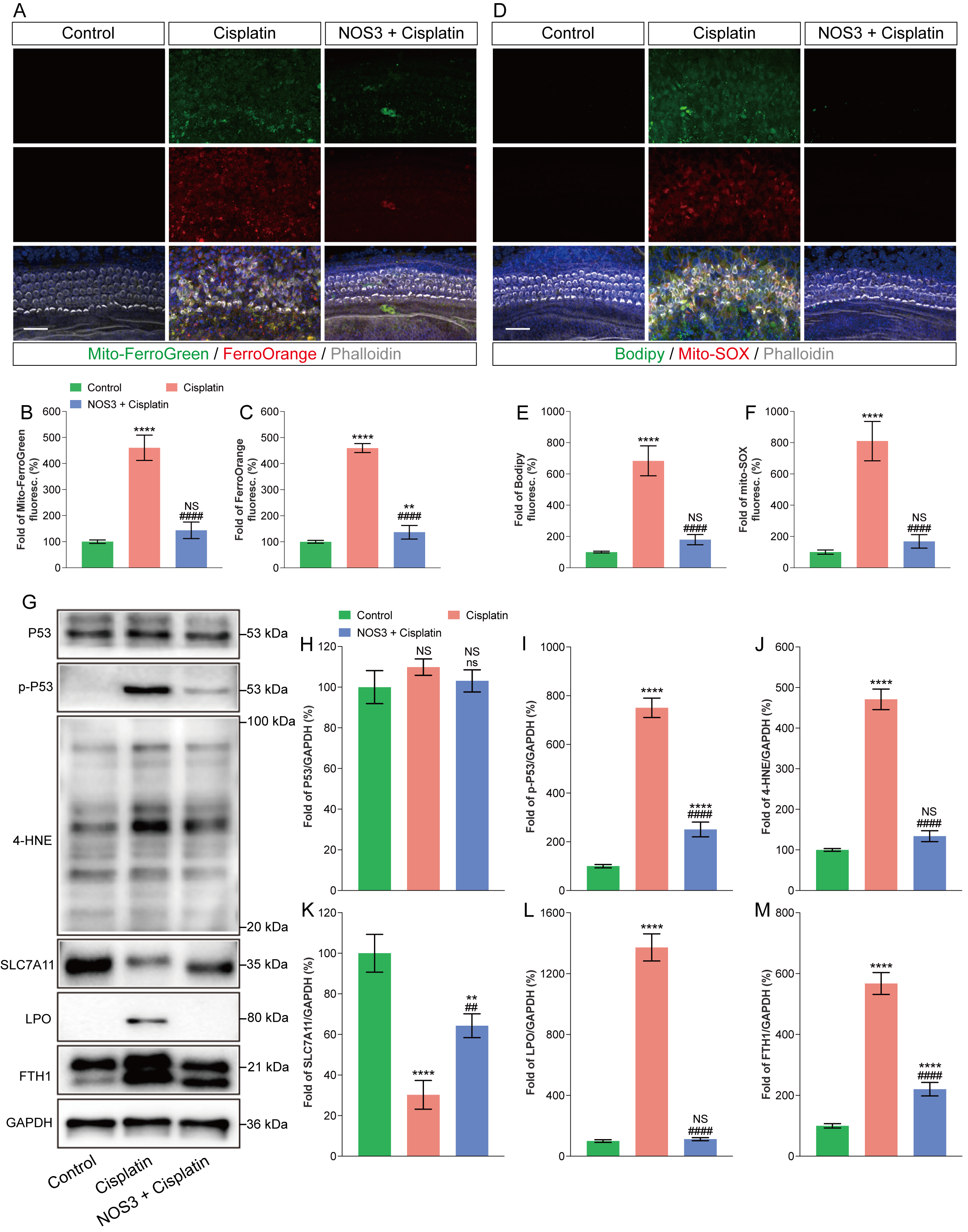


**Supplementary Figure 6. NOS3 reduces cisplatin‑induced ferroptosis in hair cells.**

**(A)** Representative immunofluorescence images of the middle turn in cultured cochlear basilar membrane explants after 48 h of treatment in Control, Cisplatin, and Cisplatin + NOS3 groups. Hair cells (labeled with phalloidin, gray) are colocalized with Mito‑FerroGreen (green; mitochondrial labile iron) and FerroOrange (red; cytosolic labile iron). Scale bars = 30 μm. **(B, C)** Quantification of mean fluorescence intensity for Mito‑FerroGreen (B) and FerroOrange (C) within the hair‑cell region shown in (A). **(D)** Representative immunofluorescence images of the middle turn in explants cultured for 48 h under the indicated treatments. Hair cells (phalloidin, gray) are colocalized with BODIPY 581/591 (green; lipid peroxidation) and MitoSOX Red (red; mitochondrial superoxide). Scale bars = 30 μm. **(E, F)** Quantification of mean fluorescence intensity for BODIPY (E) and MitoSOX Red (F) within the hair‑cell region shown in (D). **(G)** Western blot images of P53, phospho‑P53 (p‑P53), 4‑HNE, LPO, FTH1 and SLC7A11 in cochlear explants cultured for 24 h under the indicated treatments. GAPDH served as loading control. **(H–M)** Quantification of relative protein expression for P53 (H), p‑P53 (I), 4‑HNE (J), LPO (K), FTH1 (L) and SLC7A11 (M), normalized to the Control group. Data are presented as mean ± SEM. ^*^p < 0.05, ^**^p < 0.01, ^***^p < 0.001, ^****^p < 0.0001 vs. Control group; one‑way ANOVA with Bonferroni post‑hoc test, n = 6.


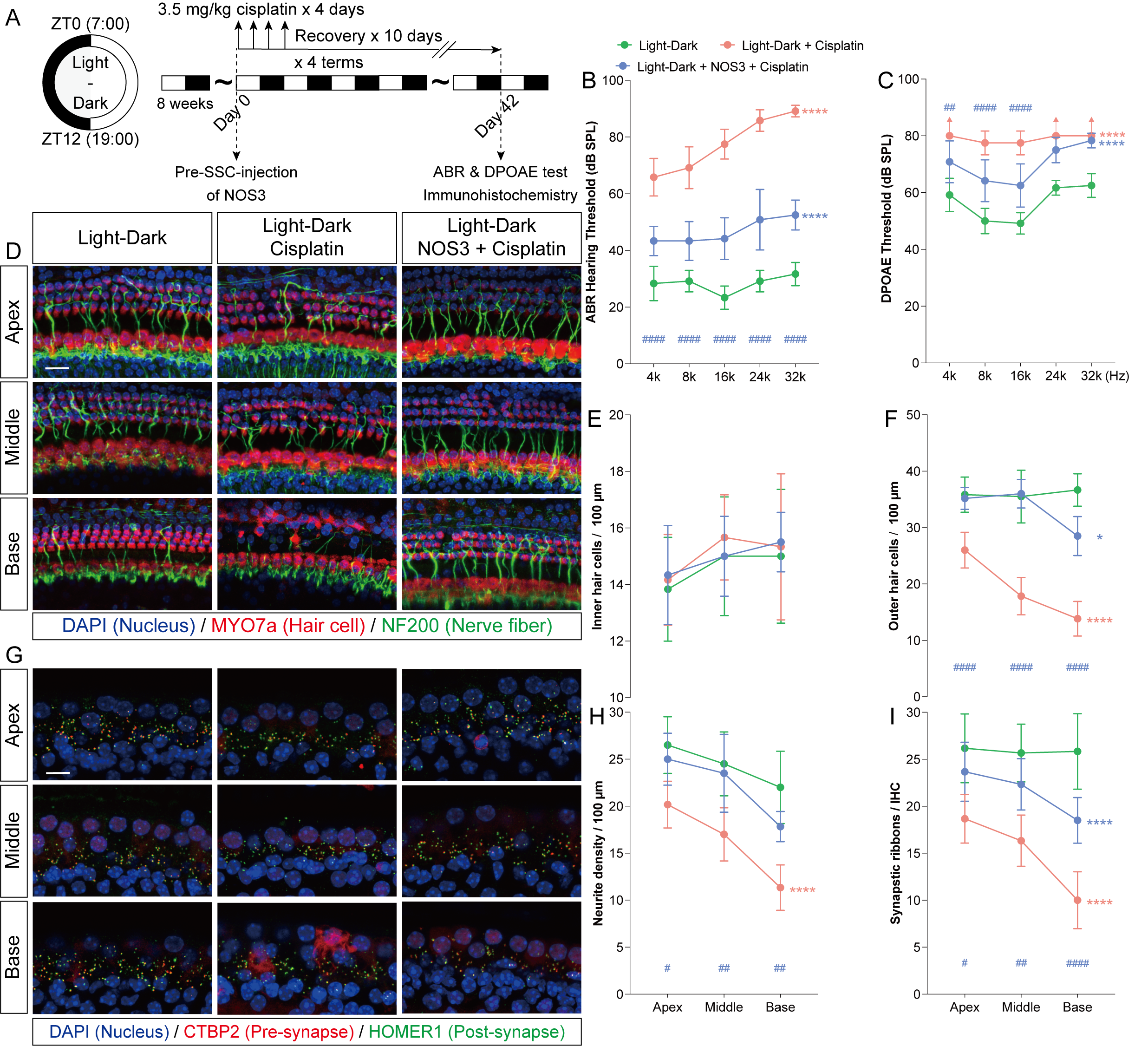


**Supplementary Figure 7. NOS3 protects against cisplatin‑induced hearing loss in mice under normal circadian conditions.**

**(A)** Schematic of cisplatin chemotherapy combined with NOS3 treatment in mice with intact circadian rhythms. **(B, C)** Post‑treatment auditory brainstem response (ABR) and distortion‑product otoacoustic emission (DPOAE) thresholds in Control, Cisplatin, and Cisplatin + NOS3 groups. **(D)** Representative immunofluorescence images of apical, middle, and basal turns of the cochlear basilar membrane after treatment. Hair cells are labeled with Myosin VIIa (red), auditory nerve fibers with neurofilament (green), and nuclei with DAPI (blue). Scale bars = 20 μm. **(E)** Representative immunofluorescence images of the ribbon‑synapse region in inner hair cells across cochlear turns after treatment. Presynaptic ribbons are labeled with Ctbp2 (red), postsynaptic densities with Homer1 (green), and nuclei with DAPI (blue). Scale bars = 10 μm. **(F–I)** Quantification of inner‑hair‑cell survival (F), outer‑hair‑cell survival (G), auditory‑nerve‑fiber terminal density (H), and intact ribbon‑synapse density (I) in apical, middle, and basal cochlear turns after treatment. Data are presented as mean ± SEM. ^*^p < 0.05, ^**^p < 0.01, ^***^p < 0.001, ^****^p < 0.0001 vs. Control group; one‑way ANOVA with Bonferroni post‑hoc test, n = 6.


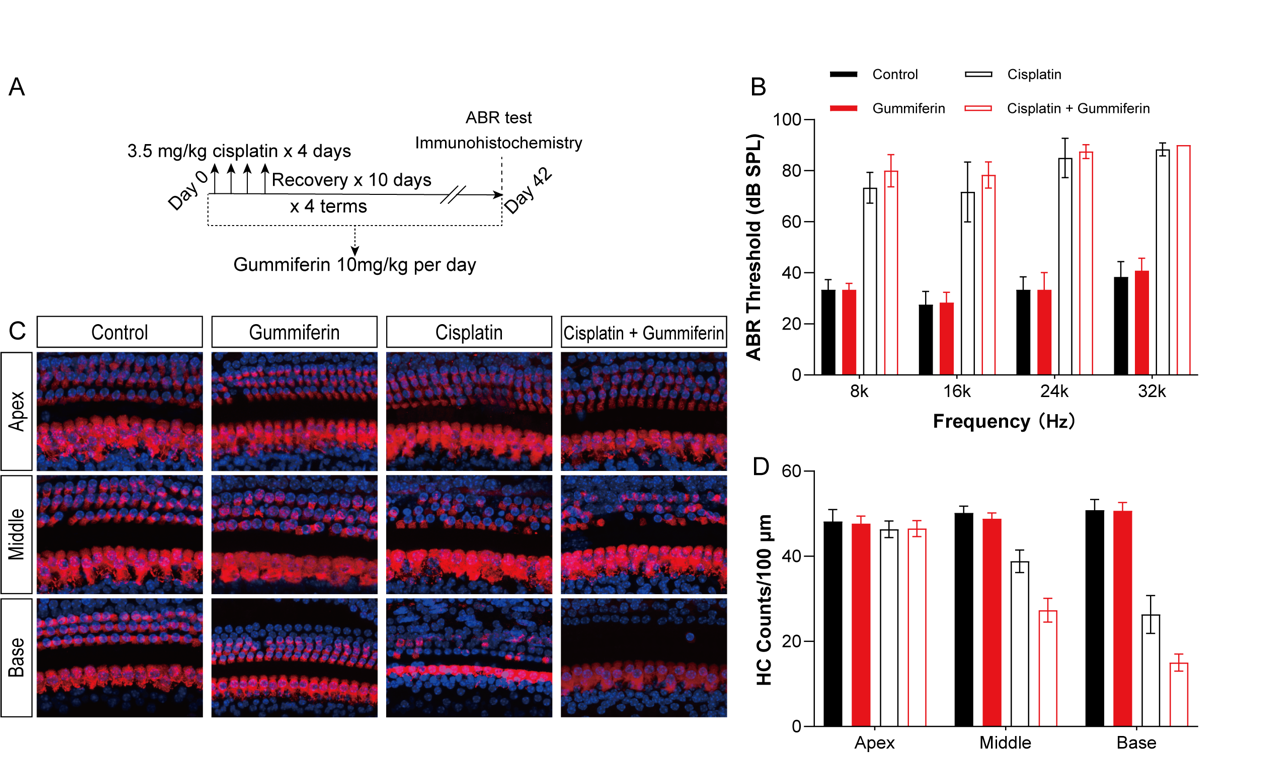


**Supplementary Figure 8. Gummiferin-induced somnolence exacerbates cisplatin-induced ototoxicity.**

**(A)** Schematic of cisplatin chemotherapy in mice with Gummiferin treatment for somnolence attack. **(B)** Post‑treatment ABR thresholds in Control, Gummiferin, Cisplatin, and Cisplatin + Gummiferin groups. **(C)** Representative immunofluorescence images of apical, middle, and basal turns of the cochlear basilar membrane after treatment. Hair cells are labeled with Myosin VIIa (red). **(D)** Quantification of hair‑cell survival in apical, middle, and basal cochlear turns after treatment. Data are presented as mean ± SEM, n = 6.


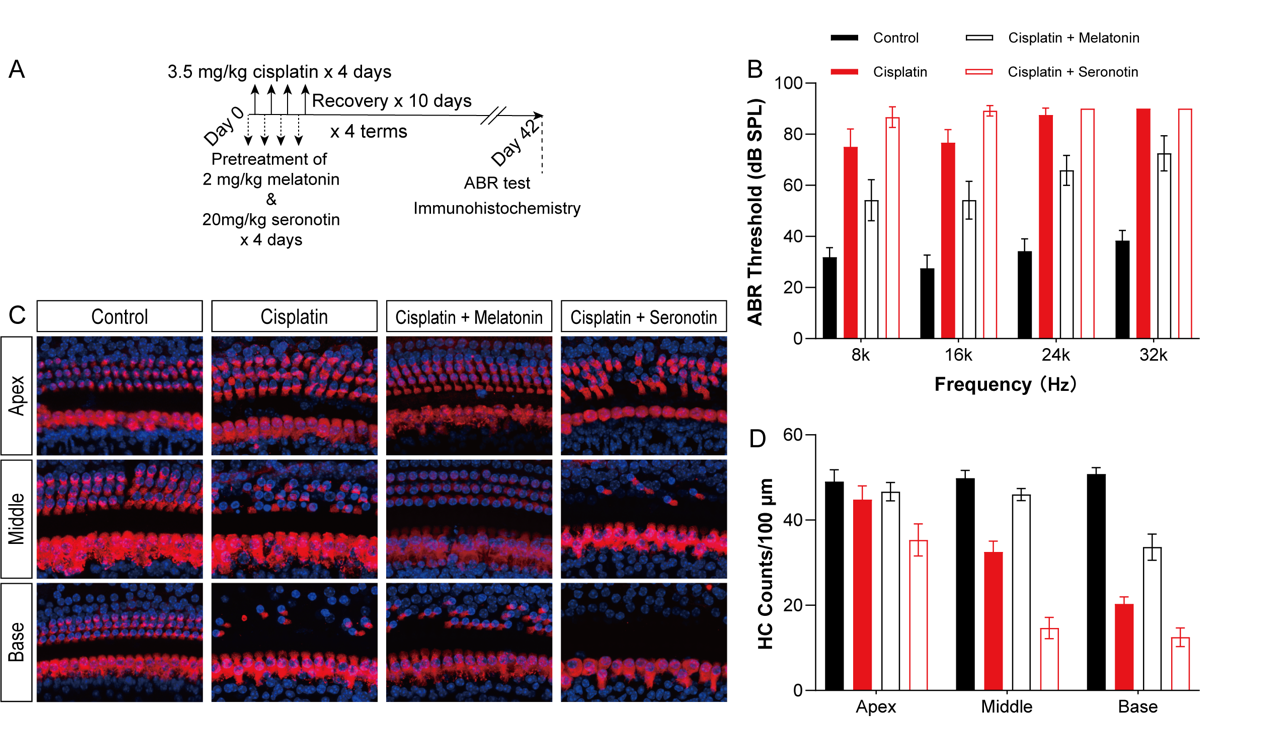


**Supplementary Figure 9. Low-dose melatonin still confers protection against cisplatin-induced ototoxicity.**

**(A)** Schematic of experimental design combining melatonin or serotonin intervention with cisplatin chemotherapy in mice, the concentrations of melatonin and serotonin were set the ELISA array in Figure 1 F and G. **(B)** Post‑treatment ABR thresholds in Control, Cisplatin, Cisplatin + Melatonin, and Cisplatin + Serotonin groups. **(C)** Representative immunofluorescence images of apical, middle, and basal turns of the cochlear basilar membrane after treatment. Hair cells are labeled with Myosin VIIa (red). **(D)** Quantification of hair‑cell survival in apical, middle, and basal cochlear turns after treatment. Data are presented as mean ± SEM, n = 6.


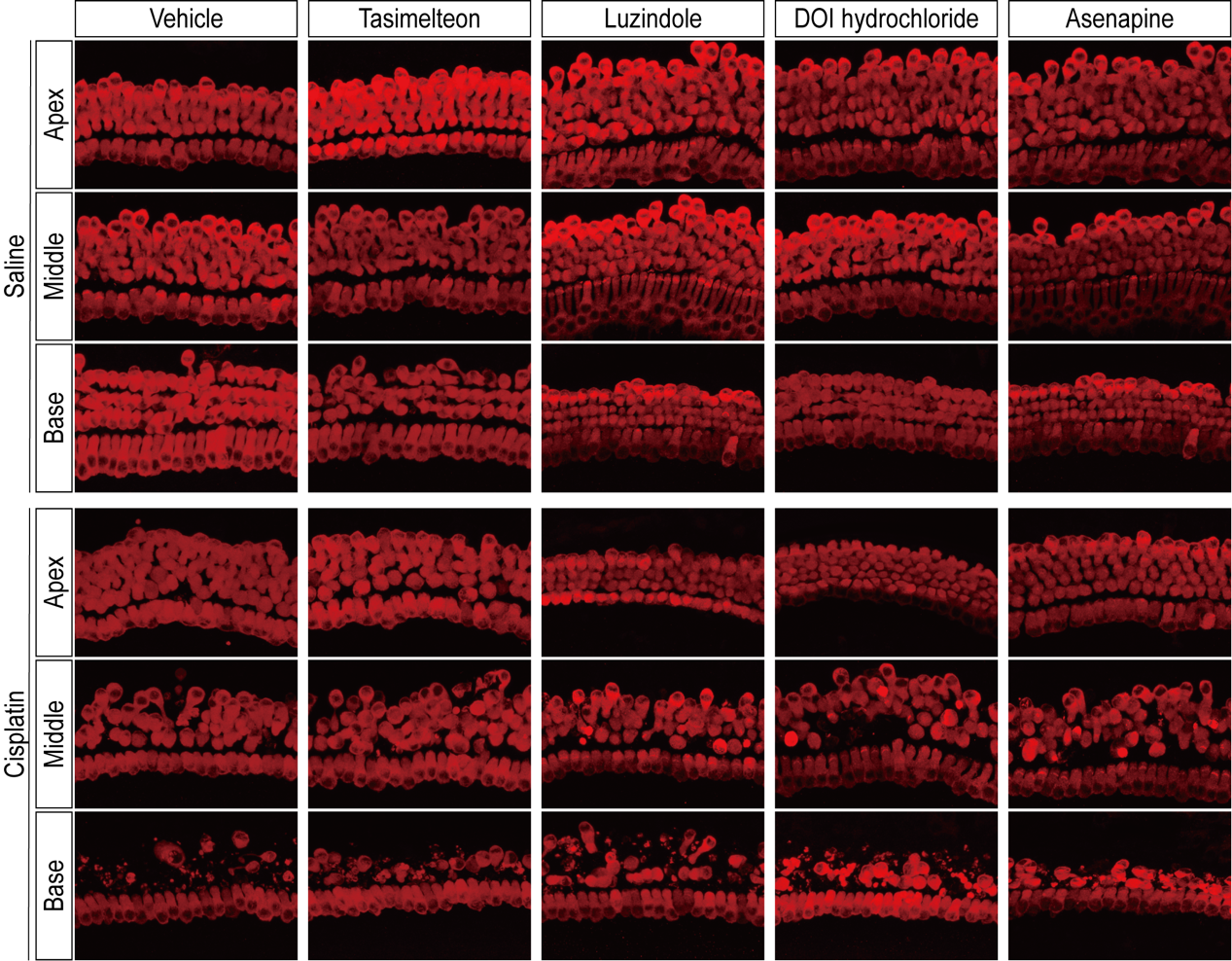


**Supplementary Figure 10. Melatonin and serotonin receptor modulators fail to inhibit cisplatin-induced hair cell damage *in vitro*.**

Representative immunofluorescence images of cochlear explants after 72 h of treatment with cisplatin, tasimelteon (melatonin receptor agonist), luzindole (melatonin receptor antagonist), DOI hydrochloride (serotonin receptor agonist), Asenapine (serotonin receptor antagonist). Hair cells are labeled with Myosin VIIa (red).


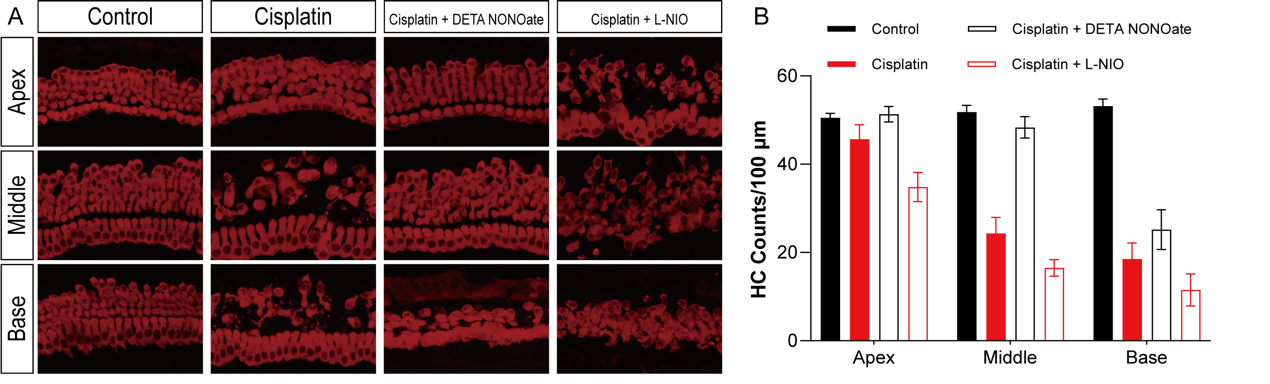


**Supplementary Figure 11. Increasing nitric oxide concentration in the culture environment alleviates cisplatin-mediated hair cell damage *in vitro*.**

**(A)** Representative immunofluorescence images of apical, middle, and basal turns of the cochlear basilar membrane in Control, Cisplatin, Cisplatin + DETA NONOate (Exogenous nitric oxide donors‌), and Cisplatin + L-NIO (nitric oxide synthase inhibitor) groups after treatment. Hair cells are labeled with Myosin VIIa (red). **(B)** Quantification of hair‑cell survival in apical, middle, and basal cochlear turns after treatment. Data are presented as mean ± SEM, n = 6.


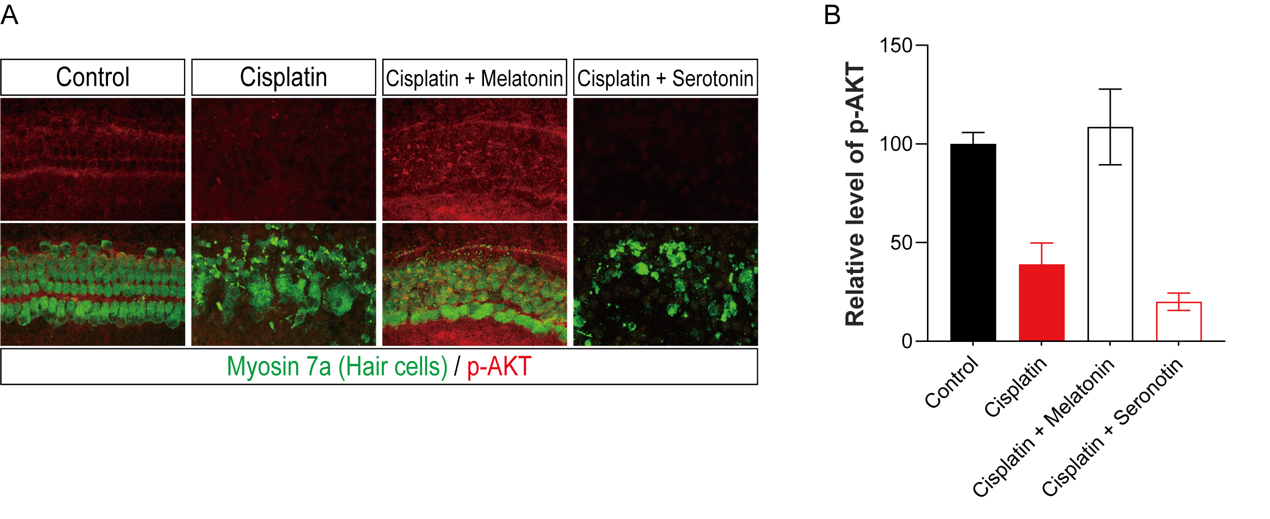


**Supplementary Figure 12. Melatonin and serotonin modulate the critical NOS3 phosphorylating kinase p-Akt.**

**(A)** Representative immunofluorescence images of the middle turn of cochlear basilar membrane explants in Control, Cisplatin, Cisplatin + Melatonin, and Cisplatin + Serotonin groups cultured for 48 h. Parvalbumin (green) labels hair cells, showing colocalization with p-AKT (red). **(B)** Quantitative analysis of the mean fluorescence intensity of NOS3 and active (phosphorylated) NOS3 within the hair‑cell region from panel (A). Data are presented as mean ± SEM, n = 6.


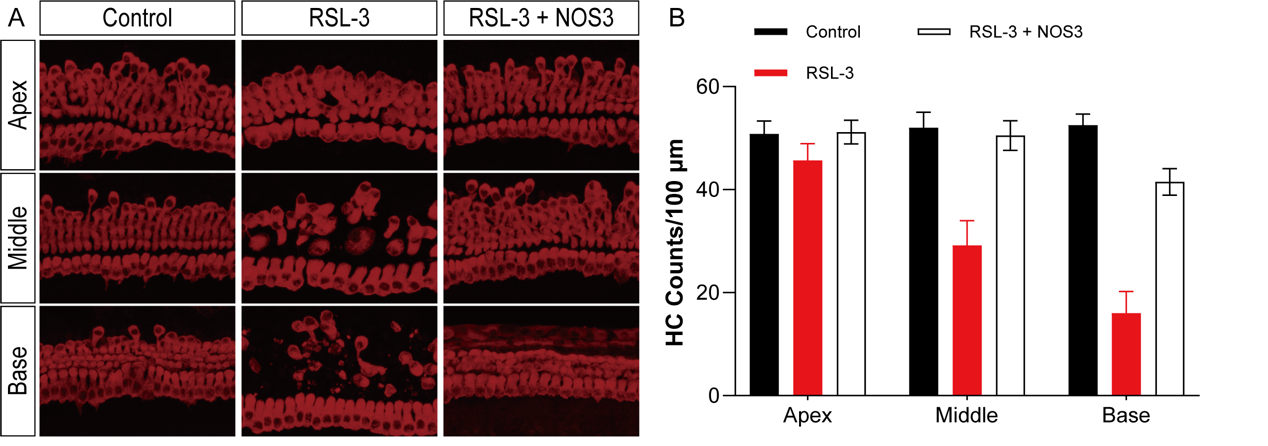


**Supplementary Figure 13** **revision. NOS3 alleviates RSL-3-mediated hair cell damage *in vitro*.**

**(A)** Representative immunofluorescence images of apical, middle, and basal turns of the cochlear basilar membrane in Control, RSL-3 (ferroptosis inducer), RSL-3 + NOS3 groups after treatment. Hair cells are labeled with Myosin VIIa (red). **(B)** Quantification of hair‑cell survival in apical, middle, and basal cochlear turns after treatment. Data are presented as mean ± SEM, n = 6.
